# Signals of Ancestry-Specific Selection in Gentle Africanized Honey Bees

**DOI:** 10.1093/gbe/evaf217

**Published:** 2025-11-18

**Authors:** Maximilian Genetti, Russell Corbett-Detig

**Affiliations:** Genomics Institute, University of California, Santa Cruz, CA 95064, USA; Department of Biomolecular Engineering, University of California, Santa Cruz, CA 95064, USA; Genomics Institute, University of California, Santa Cruz, CA 95064, USA; Department of Biomolecular Engineering, University of California, Santa Cruz, CA 95064, USA

**Keywords:** admixture, hybridization, selection, population genomics, adaptive introgression, aggression

## Abstract

Understanding the genetic basis of adaptive responses to environmental and human mediated pressures is a central concern in evolutionary biology. Population admixture, a process wherein genetically differentiated populations interbreed, is increasingly recognized as a source of genetic material driving rapid evolutionary responses. Honey bees from Puerto Rico are a phenotypically distinct population of Africanized honey bees with demonstrably lower levels of aggression than other Africanized populations. The Puerto Rican honey bee population represents a dynamic system that has experienced both environmental and human-mediated selective pressures over a short period of time marked by a significant influx of genetic variation from mainland Africanized honey bees, which has notably influenced the genetic makeup of the local populations. In this study we detail the current population structure of the Puerto Rican honey bees, how this differs from a mainland population, and regions of the genome that have signals of ancestry-specific selection. To distinguish loci undergoing ancestry-specific selection, we use tools that co-estimate local ancestry and the strength of selection at loci across the genome. We further detail the genes and pathways highlighted through gene ontology (GO) enrichment analysis. Overall, our results suggest that the local pressures on Puerto Rico honey bee behavior may have induced significant changes favoring alleles linked to different ancestries at loci and pathways involved in neuronal development, behavior, and mating among others. Our analysis demonstrates that approaches that explicitly model selection on local ancestry may be valuable tools for understanding evolution in admixture zones.

Significance In 1957, a highly aggressive population of honey bees escaped from a Brazilian breeding program crossing European domestic honey bee stocks with a South African subspecies to produce a subtropical-adapted honey bee. The escaped hybrids, later known as Africanized honey bees (AHBs), rapidly spread throughout South and Central America, eventually reaching North America. This study explores the evolution of Puerto Rican AHBs, which became significantly less aggressive than their mainland counterparts. Using local ancestry inference (LAI) methods, we identified 14 regions with signatures of selection favoring European-like ancestry and involved in key biological processes, including neural development and behavioral regulation. Notably, these regions overlap with genes influencing behavior, such as serotonin and glutamate receptors. Our findings suggest that European-derived haplotypes play a crucial role in the reduction of aggression, illustrating how genetic admixture may contribute to rapid adaptation and complex behavioral traits in honey bee populations.

## Introduction

Admixture is increasingly thought to be a major source of new genetic variation in natural populations (Cuadros-Espinoza et al. 2022; Lucena-Perez et al. 2024) and that variation introduced through admixture can produce adaptive phenotypic outcomes (Racimo et al. 2015; Oziolor et al. 2019). Although selection on newly evolved and standing genetic variation within populations has been extensively studied (McCoy and Akey 2017; Lai et al. 2019; Ji et al. 2020), the signature of adaptation resulting from ancestry-specific selection, or introgression when there is selection on the minor ancestry, is only beginning to be explored in detail (Huerta-Sánchez et al. 2014; Schumer et al. 2018; Rinker et al. 2020; Ayala et al. 2023). Importantly, selection on genetic variation introduced through admixture has a characteristic signal wherein many distinct haplotypes from a donor population may contain the same or functionally equivalent adaptive variant(s) (Setter et al. 2020; Shchur et al. 2020; Svedberg et al. 2021). Analyses that specifically focus on this signature are crucial for understanding the genomic consequences and prevalence of adaptation resulting from admixed genetic variation.

Selection on variation contributed by admixture from ancestral populations can be difficult to detect, but the distinctive pattern left on the local ancestry tracts surrounding adaptive variation can reveal it (Shchur et al. 2020; Svedberg et al. 2021; Ayala et al. 2023). Due to their rapid increase in frequency, adaptive haplotypes tend to have longer surrounding segments of non-recombined ancestry compared to what's expected under neutrality. This scenario resembles a soft selective sweep, where an adaptive allele is present within multiple haplotypes, but differs in that a haplotype introduced via introgression carries even greater genetic diversity from the same source population and may still undergo selection similar to that seen in hard selective sweeps (Santiago and Caballero 2005; Tang et al. 2007; Garud 2023; Panigrahi et al. 2023).

In 1957, 26 African honey bee queens (*Apis mellifera scutellata*) escaped from an experimental breeding program south of São Paulo, Brazil, leading to extensive interbreeding with Domestic European honey bees (*A. m. **subspp.***) throughout the Americas (Pinto et al. 2005; Francoy et al. 2009; Nelson et al. 2017; Carpenter and Harpur 2021). When African honey bees were introduced into the Americas, Brazil's commercial honey bee population originated from at least two distinct European sources: the M-lineage (including *A. m. mellifera* and *A. m. iberiensis)*, and the C-lineage (including *A. m. ligustica* and *A. m. carnica)* (Harpur et al. 2020). The resulting hybrid population, known as Africanized honey bees (AHBs), are highly aggressive, often able to outcompete European honey bees, and expand through both queen and Male migration (Smith et al. 1989). Shortly after their emergence, AHBs spread North, reaching the continental United States in 1993 and Puerto Rico in 1994 (Cox 1994) where at least eight honey bee subspecies from three of the five major global lineages had been previously introduced, with the modern domestic population consisting primarily of the C and M lineages and the A and O lineages to a lesser extent (Carpenter and Harpur 2021).

Unusual among domesticated species, honey bees do not show signs of domestication bottlenecks, but actually show genetic diversity beyond that of ancestral wild populations (Harpur et al. 2012; Oldroyd 2012). Despite descending from a limited number of African queens, Africanized bees in South America maintain levels of genetic variation comparable to African subspecies (Schneider et al. 2004). Genetic ancestry studies reveal that the tropical and subtropical Americas are largely populated by bees of high African ancestry with stable hybrid zones between predominantly European and African bees spanning similar latitudes in both North and South America (Calfee et al. 2020). This hybridization offers an opportunity to explore various Africanized honey bee subpopulations for signs of ancestry-specific selection shaping their adaptation to the Americas, including near their initial introduction (Nelson et al. 2017), high altitudes (Everitt et al. 2023), hybrid clines (Calfee et al. 2020), and the isolated island environment of Puerto Rico (Avalos et al. 2017)

Shortly after the arrival of AHBs to Puerto Rico in 1993, the local government enacted a plan to rapidly eliminate aggressive hives (Senado de Puerto Rico 1999). In 2006, Africanized honey bee colonies in Puerto Rico were discovered that had undergone a drastic reduction in aggression relative to mainland AHBs, with behaviors that closely resemble that of European honey bees. In line with the goals of the original Brazilian breeding program, these honey bees also maintained other traits of the African honey bee, such as resistance to ectoparasites (e.g. Varroa mites) and small size (Rivera-Marchand et al. 2012). Avalos et al. (2017) reports the occurrence of a soft selective sweep on standing variation within the AHBs of Puerto Rico, leading to the observed “gentle” Africanized honey bees (gAHB) phenotype. However, selection on alleles linked to local ancestry may not result in the fixation of selected alleles or on the extended haplotype homozygosity characteristic of selective sweeps. In particular, given that European honey bees are less aggressive toward humans, we may expect to find additional genomic regions that are selected within gentle populations that broadly favor European haplotypes.

Here, we apply an analysis method that explicitly models local ancestry during admixture to search for ancestry-specific selection in gAHBs and contrast it with an aggressive Africanized honey bee population from Mesoamerica. In total, we identify 14 regions with signatures of selection for European ancestry in Puerto Rican honey bees, one of which is also apparent within the Mesoamerican honey bee population. Genes contained within these regions are enriched for pathways involved with tissue morphogenesis, growth regulation, cell proliferation and carbohydrate metabolism. Of particular interest is the presence of major regulators of neural development, two G-protein-coupled receptors (GPCRs) for neurotransmitters, the gamma subunit of the heterotrimeric G protein, and a probable ras GTPase-activating protein. The GPCRs include the serotonin receptor 5-HT7, and a subunit of the glutamate receptor NMDA. We observe only moderate overlap with prior population genetic analysis that search for classic signatures of natural selection within this population, emphasizing the potential importance of local-ancestry-aware methods of detecting ancestry-specific selection.

## Results

### Population Structure

We analyzed genomic data from Puerto Rican (PR; *n* = 30) and Mesoamerican (MA; *n* = 25).

**Fig. 1.**
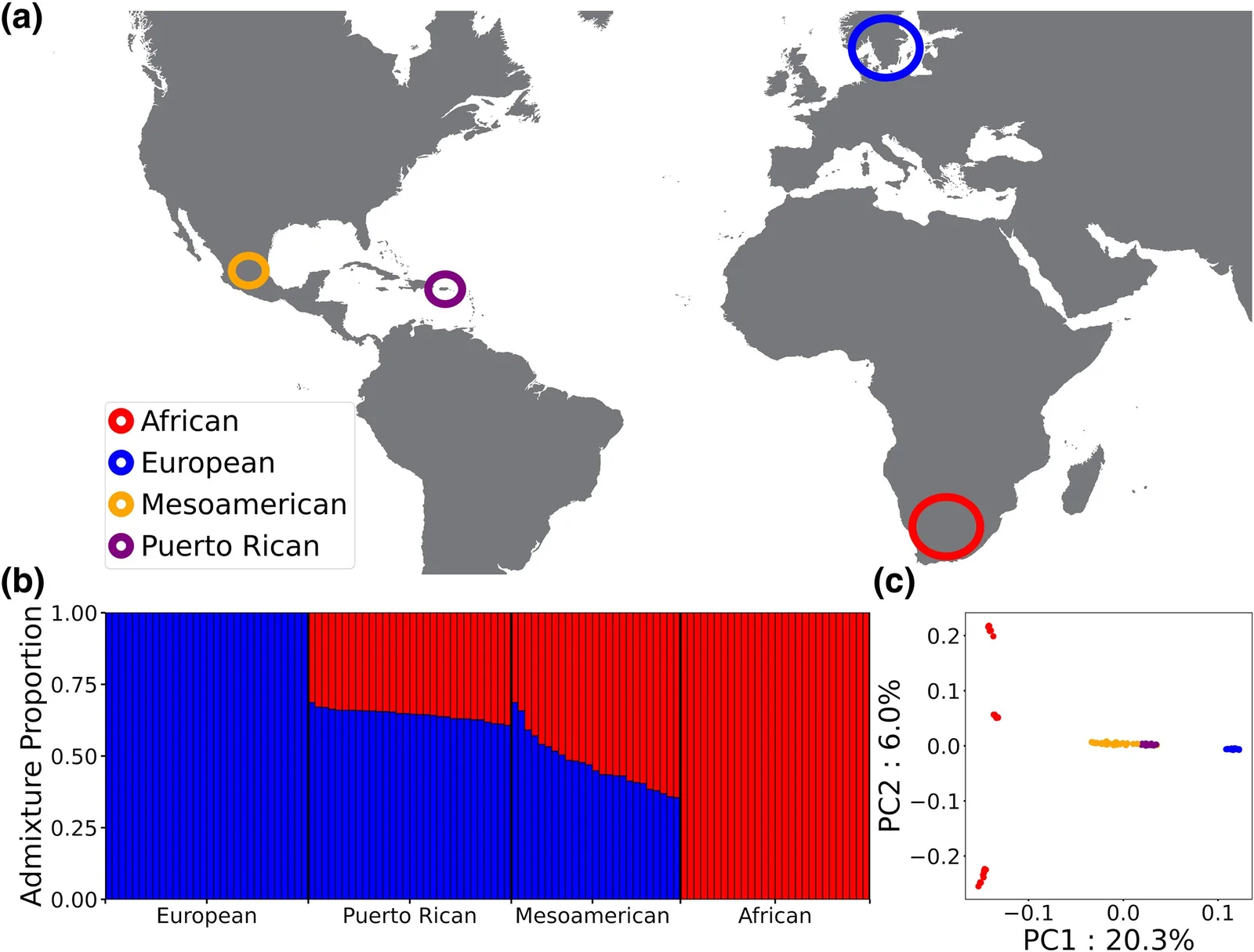
Overview of sampling locations and population structure. a) The approximate sampling locations of the European, Puerto Rican, Mesoamerican and African honey bees. b) ADMIXTURE (K = 2, F_st_ of 0.368) results of European, Puerto Rican, Mesoamerican and African honey bees. Puerto Rican honey bees had an average of 0.646 European-like ancestry. Mesoamerican honey bees had an average of 0.471 European-like ancestry. c) Principal components 1 and 2 of PCAngsd analysis of the genotype data. The 4 populations separate along the first principal component (PC1) which explains 20.3% of the variance, with African and European samples at opposite extremes and Puerto Rican and Mesoamerican samples intermediate and overlapping. The second principal component (PC2) which explains 6.0% of the variance, demonstrates comparatively limited structure within the African honey bee samples.

Africanized honey bee populations, along with European (EUR; *n* = 30) and African (AFR; *n* = 28) reference panels representing the ancestral populations (Table S1). ADMIXTURE and PCAngsd analyses suggest that differentiation between the ancestral European and African lineages and subsequent admixture is the primary source of population structure in these samples with F_st_ of 0.368 (Fig. 1). The population structure analyses reveal a relatively homogeneous population within Puerto Rico that has consistent ADMIXTURE estimates with an average proportion of 0.646 European ancestry (Fig. 1b, Fig. S1a). The Mesoamerican population has more varied ancestry proportions with an average of 0.471 (Fig. 1b, Fig. S1a). PCAngsd (Fig. 1c, Fig. S1b) provides approximately concordant results. PCAngsd further demonstrate that a majority of the variation within these samples is explained by the variation between the European and African populations captured in part by the first principal component and supports their use as panels representative of the ancestral populations of the AHBs (Fig. 1c, Fig. S1b). PCAngsd does suggest a degree of variation within the African population as seen with the second principal component, but this only explains a relatively minor amount of the total variance at 6.0% (Fig. 1c).

**Fig. 2.**
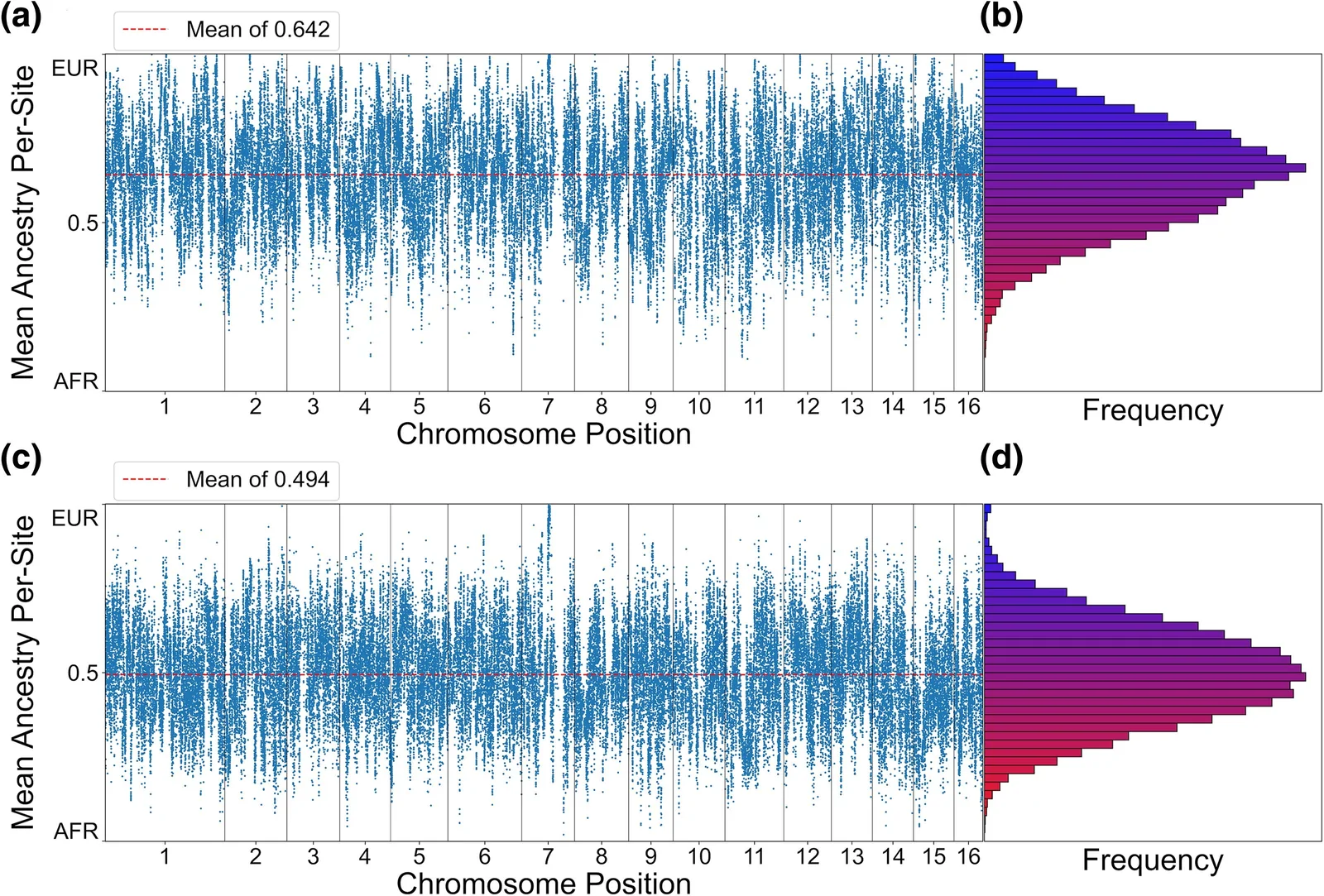
Local ancestry inference and site-specific ancestry frequency spectrum. a) Genome-wide local ancestry estimates of the Puerto Rican population is shown as the mean posterior probability for all samples in a given population at each ancestry informative maker (AIM) as estimated using Ancestry_HMM. Vertical black lines indicate chromosome boundaries. b) Ancestry_HMM results for Puerto Rican population represented as a histogram of the mean posterior probability per site across all individuals. c) The local ancestry of the Mesoamerican population is shown as the mean posterior probability at each AIM as estimated using Ancestry_HMM. Vertical black lines indicate chromosome boundaries. d) Ancestry_HMM results for Mesoamerican population represented as a histogram of the mean posterior probability per site across all individuals.

The population substructure observed using the local ancestry inference method Ancestry_HMM obtained largely concordant ancestry fraction estimates with our prior methods, but also demonstrates substantial variation in local ancestry frequencies along the genome (Fig. 2). The mean posterior probability across the genome for European ancestry is 0.642 and 0.494 for the Puerto Rican and Mesoamerican populations respectively (Fig. 2a and c). These results are largely consistent with our prior ADMIXTURE analysis (0.646, and 0.471, respectively). Using Ancestry_HMM, the number of generations since admixture is estimated at 74 and 125 for Puerto Rican and Mesoamerican honey bees respectively. The difference in estimates might also be consistent with a recent and significant pulse of European ancestry during the colonization of Puerto Rico, which would introduce larger European haplotypes and reduce the apparent number of generations estimated since admixture has begun. Additionally, given the high variance in overall ancestry fraction of Mesoamerican sample, a single admixed population may not accurately capture the population dynamics. These values are approximately consistent with previously reported estimates of the number of generations since this admixture event began in 1957 and the time of collection in 2015 (Avalos et al. 2017; Calfee et al. 2020). The estimated generations per year, 1.28 and 2.16, are faster than the generation time for domestic honey bees, but are consistent with the previously reported lower generation time of AHBs (Wallberg et al. 2014; Calfee et al. 2020).

### Signatures of Selection

Our analysis using AHMM-S detected selection for both African and European ancestry in North American AHBs, with some genomic regions shared and unique to honey bee populations in Mesoamerica and Puerto Rico (Table 1, Table S2, and Fig. S2). We identified 14 regions across 7 chromosomes in Puerto Rican honey bees and 4 regions across 3 chromosomes in Mesoamerican honey bees with significant signals of selection favoring European ancestry. In contrast we found fewer sites with evidence of ancestry-specific selection on African ancestry. We identified 7 regions across 3 chromosomes in Puerto Rican honey bees and 2 regions across 2 chromosomes in Mesoamerican honey bees with significant signals of selection favoring African ancestry. Among these, only one is shared, with a region on chromosome 7 that shows significant signals of selection and near fixation for European ancestry in both Puerto Rican and Mesoamerican honey bees.

**Table 1:** Putative targets of selection

Population	Ancestry	Peak	Chromosome	Position	EUR ancestry proportion	Log-likelihood Ratio	Proximal GeneIDs	Proximal gene descriptions
PR	AFR	PF1	chr5	7,767,124	0.144	25.578	409818	sodium/calcium exchanger 1-like
PR	AFR	PF2	chr10	5,909,139	0.274	34.392	…	…
PR	AFR	PF3	chr10	6,057,267	0.304	32.464	410229	toll-like receptor 6
PR	AFR	PF4	chr10	8,141,101	0.271	45.664	724333	beta-1-syntrophin
PR	AFR	PF5	chr11	4,256,369	0.119	31.638	725924	leucine-rich repeat-containing protein 24
PR	AFR	PF6	chr11	5,642,990	0.095	33.638	…	…
PR	AFR	PF7	chr11	6,101,187	0.202	29.921	102654083	centrosome-associated zinc finger protein CP190
PR	EUR	PR1	chr1	2,929,896	0.868	25.687	724238	paired box protein Pax-6
PR	EUR	PR2	chr1	15,255,347	1.000	40.3	107963975	uncharacterized LOC107963975
PR	EUR	PR3	chr1	15,851,999	0.963	25.317	726702	serotonin receptor 7
PR	EUR	PR4	chr1	17,483,461	0.945	24.732	410009	cadherin-related tumor suppressor
PR	EUR	PR5	chr1	22,337,326	0.927	43.008	726766	endoribonuclease Dicer
PR	EUR	PR6	chr1	27,886,171	0.951	26.215	410775	laminin subunit alpha-1
PR	EUR	PR7	chr3	10,566,232	0.882	26.81	100578771	probable Ras GTPase-activating protein
PR	EUR	PR8	chr4	9,377,455	0.964	28.636	413423	hemicentin-2
PR	EUR	PR9	chr4	9,749,904	0.932	28.108	725627	clotting factor B
PR	EUR	PR10	chr6	20,34,269	0.899	27.609	…	…
PR	EUR	PR11	chr7	6,915,854	0.996	30.586	727091	forkhead box protein O
PR	EUR	PR12	chr7	12,200,935	0.959	26.82	412818, 100577159	NMDA receptor subunit 2, uncharacterized LOC100577159
PR	EUR	PR13	chr14	1,847,557	0.967	25.987	725453	guanine nucleotide-binding protein subunit gamma-e
PR	EUR	PR14	chr15	4,658,368	0.986	24.86	726229	uncharacterized LOC726229
MA	AFR	MF1	chr8	3,246,532	0.119	25.907	408958	nucleolar protein 4-like
MA	AFR	MF2	chr15	1,462,405	0.051	31.092	677673	odorant binding protein 14
MA	EUR	MR1	chr5	3,036,328	0.883	28.305	406073	homeobox protein prospero
MA	EUR	MR2	chr7	6,744,151	0.996	59.833	727091	forkhead box protein O
MA	EUR	MR3	chr13	8,690,069	0.895	26.64	724773	uncharacterized LOC724773
MA	EUR	MR4	chr13	8,925,953	0.874	26.769	725053	protein odd-skipped

Within Puerto Rican honey bees (PR) we see evidence for selection acting on 14 distinct regions of European ancestry (EUR) and 7 regions of African ancestry (AFR). Within Mesoamerican honey bees (MA) we see evidence for selection acting on 4 regions for European ancestry and 2 regions for African ancestry.

We asked if our AHMM-S analysis was dependent on nucleotide diversity, exon density or recombination rates (Fig. S3) around the signals of selection (Table S3). In Puerto Rican honey bees, Spearman's correlations between log-likelihood ratios and all genomic features were weak and non-significant for both European and African ancestry (|ρ| < 0.15, *P* > 0.10 for nucleotide diversity and exon density; |ρ| < 0.62, *P* > 0.06 for recombination rate), consistent with little or no correlation (Akoglu 2018). In contrast, the Mesoamerican population showed significant correlations between signals of selection and some genomic features. Signals of selection on African ancestry showed significant negative correlation with nucleotide diversity (ρ = −0.66, *P* = 0.038) and positive correlation with exon density (ρ = 0.83, *P* = 0.003). Signals of selection on European ancestry showed significant negative correlation with exon density (ρ = −0.88, *P* < 0.001) and positive correlation with recombination rate (ρ = 0.77, *P* = 0.009). The presence of correlations in the Mesoamerican population but not in Puerto Rican honey bees may reflect the more varied ancestry proportions and demographic history of the Mesoamerican population (Fig. 1b). Notably, the correlation patterns differ in direction between ancestries and features, suggesting complex interactions rather than systematic artifacts. Pearson's correlation between the likelihood ratios for each population on European and African ancestry are 0.359 and 0.407 respectively (both with *P* < 0.001). These values of *r* are relatively low and demonstrate that the signals of selection for each ancestry between the populations are only weakly correlated (Akoglu 2018), consistent with distinct selection pressures experienced in each population.

A small founding Africanized honey bee population or a significant bottleneck after introduction followed by genetic drift could lead to regions with reduced diversity of ancestral haplotypes with the appearance of ancestry-specific selection. Early reports on the Africanized honey bee population in Puerto Rico describe it to be highly aggressive (Senado de Puerto Rico 1999) indicating the loss of aggressive haplotypes occurred after introduction. Prior studies have shown that the Puerto Rican population may have experienced a genetic bottleneck, but that it was extended over a decade (Avalos et al. 2017). Through measuring ancestry-specific haplotype diversity across the genome, we demonstrate a modest (∼25%) difference in haplotype diversity in the Puerto Rican population relative to the mainland Mesoamerican population and that it is not ancestry specific (Table 2). While this does not completely exclude a potential impact from a demographic process, the lack of correlation of signals of selection with *π* or association with a loss in haplotype diversity for either ancestry type suggests that demography is not a major confounder for these signals of selection. However, a more detailed analysis and enhanced sample may reveal complicated demographic processes that include the effects of population founding in Brazil, expansion through south and central America and ultimately colonization of Puerto Rico.

**Table 2:** Haplotype diversity

Population	Ancestry	Mean	Standard Deviation
PR	EUR	2.666	1.068
PR	AFR	2.598	1.114
MA	EUR	3.503	1.659
MA	AFR	3.61	1.829

The mean number of individual haplotypes observed in 200 bp windows sampled across Puerto Rican (PR) and Mesoamerican (MA) honey bees in regions of shared African (AFR) and European (EUR) ancestry. There is reduced haplotype diversity in PR honey bees relative to the mainland MA population for both EUR and AFR haplotypes.

### Overlap With Prior Analyses

One previous study of these Africanized honey bee samples searched for signatures of soft sweeps within the Puerto Rico population which relies on a different methodology and has little overlap with our study (Avalos et al. 2017). We expect that the difference stems in large part from the distinct signatures of selection that local-ancestry aware methods such as ours are designed to detect relative to more traditional soft and hard sweep models. There is one signal of selection on chromosome 14 proximal to the gamma subunit of the heterotrimeric G protein (GeneID:725453) in our results that overlaps with a reported region in the prior study proximal to the same gene, reported as GB48472. The differences in our findings could result from the use of different parental panels, assemblies, and the sensitivity of the composite selection signal used in the prior study to the demographic history of the admixed population, i.e. population bottlenecks (Panigrahi et al. 2023). Nonetheless, the relatively modest overlap strongly implies that signatures of selection on local ancestry are a valuable complement to more traditional scans for selection.

### GO Enrichment

GO enrichment analysis identified several commonalities among the genes our analysis identified as putative targets of natural selection. In particular, we see enrichment for eleven pathways in Puerto Rican honey bees and seven within Mesoamerican honey bees. Within Puerto Rican honey bees, seven significantly enriched GO-Terms (GO:0035332, GO:0008285, GO:0009888, GO:0040008, GO:0098609, GO:0042067, GO:0007156) are involved with tissue or organ morphogenesis, growth regulation, or cell proliferation and four significantly enriched GO-Terms (GO:0051716, GO:0023052, GO:0007154, GO:0007165) are involved with signaling (Table 3). Within Mesoamerican honey bees there is enrichment for seven pathways (GO:0008285, GO:0000122, GO:0045944, GO:0006357, GO:0016358, GO:0048667, GO:0048813) which are related to the regulation of gene expression and dendrite development (Table 3).

**Table 3:** GO enrichment analysis

Population	GO-Term	*P*-value	Fold Enrichment	GeneIDs
PR	GO:0035332	9.02E-04	53.422	410009, 413423
PR	GO:0008285	2.00E-03	23.287	410009, 727091
PR	GO:0009888	2.35E-03	64.870	410009, 410775
PR	GO:0040008	2.80E-03	37.841	410009, 727091
PR	GO:0098609	1.24E-02	25.227	410009, 413423
PR	GO:0042067	1.84E-02	18.164	410009, 413423
PR	GO:0007156	2.56E-02	23.287	410009, 413423
PR	GO:0051716	2.95E-02	4.244	726702, 100578771, 725453
PR	GO:0023052	2.98E-02	4.218	726702, 100578771, 725453
PR	GO:0007154	3.37E-02	4.079	726702, 100578771, 725453
PR	GO:0007165	3.38E-02	4.019	726702, 100578771, 725453
MA	GO:0008285	1.08E-03	36.593	406073, 727091
MA	GO:0000122	2.24E-03	10.243	406073, 727091, 725053
MA	GO:0045944	2.96E-03	7.112	406073, 727091, 725053
MA	GO:0006357	4.95E-03	5.073	406073, 727091, 725053
MA	GO:0016358	4.95E-03	13.856	406073, 727091
MA	GO:0048667	4.95E-03	11.509	406073, 727091
MA	GO:0048813	4.95E-03	12.630	406073, 727091

Results of GO enrichment analysis for Biological Processes associated with genes proximal to sites with signs of selection for European Ancestry. There are 11 terms enriched in Puerto Rican honey bees and 7 in Mesoamerican honey bees

### Genes Consistent With Natural Selection

We identified a number of genes in close proximity of these signals that may have functional roles in Puerto Rican honey bees and make compelling candidates for the action of natural selection on alleles associated with local ancestry. Among the 14 regions in Puerto Rican honey bees with signals of selection for European ancestry, we describe 12 proximal genes that have been reported to have roles in neurodevelopment/behavior and immune response (Table S4). Below we highlight the significance of three of these genes. Two genes with interacting proteins that have broad effects on both behavior and neurodevelopment which have been reported in prior studies of *Apis* to be under selection in either expanding or admixing populations. One gene, near signals of selection in both Africanized honey bee populations, is implicated in several studies for its role in the overwintering ability of worker bees.

**Fig. 3.**
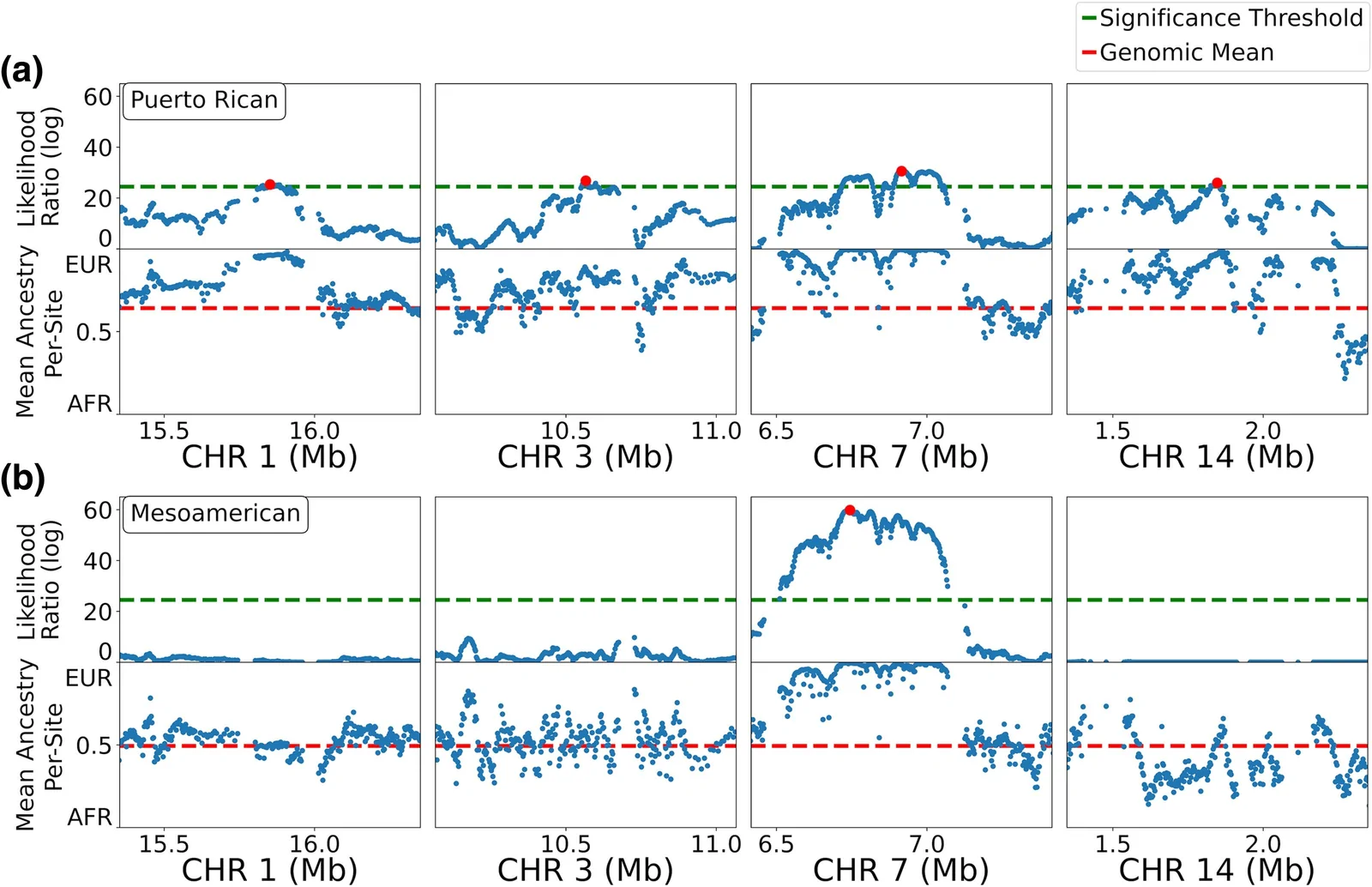
Putative targets of natural selection. a) Log-likelihood ratios from AHMM-S and mean ancestry per site from Ancestry_HMM for selection on European ancestry in Puerto Rican honey bees at sites on chromosome 1, chromosome 3, chromosome 7, and chromosome 14. b) Log-likelihood ratios from AHMM-S and mean ancestry per site from Ancestry_HMM for selection on European ancestry in Mesoamerican honey bees on the same regions of chromosome 1, chromosome 3, chromosome 7, and chromosome 14. The peak on chromosome 1 in Puerto Rican honey bees is within the mRNA transcript of the serotonin receptor 5-HT7 (GeneID:726702). The peak on chromosome 3 in Puerto Rican honey bees is within the mRNA transcript of the probable Ras GTPase-activating protein (GeneID:100578771). The region on chromosome 7 contains peaks for both Puerto Rican and Mesoamericans which are each proximal to the FOXO gene (GeneID:727091). The peak on chromosome 14 in Puerto Rican honey bees sits 249 bp upstream of the mRNA transcript of Gγ (GeneID:725453).

**Fig. 4.**
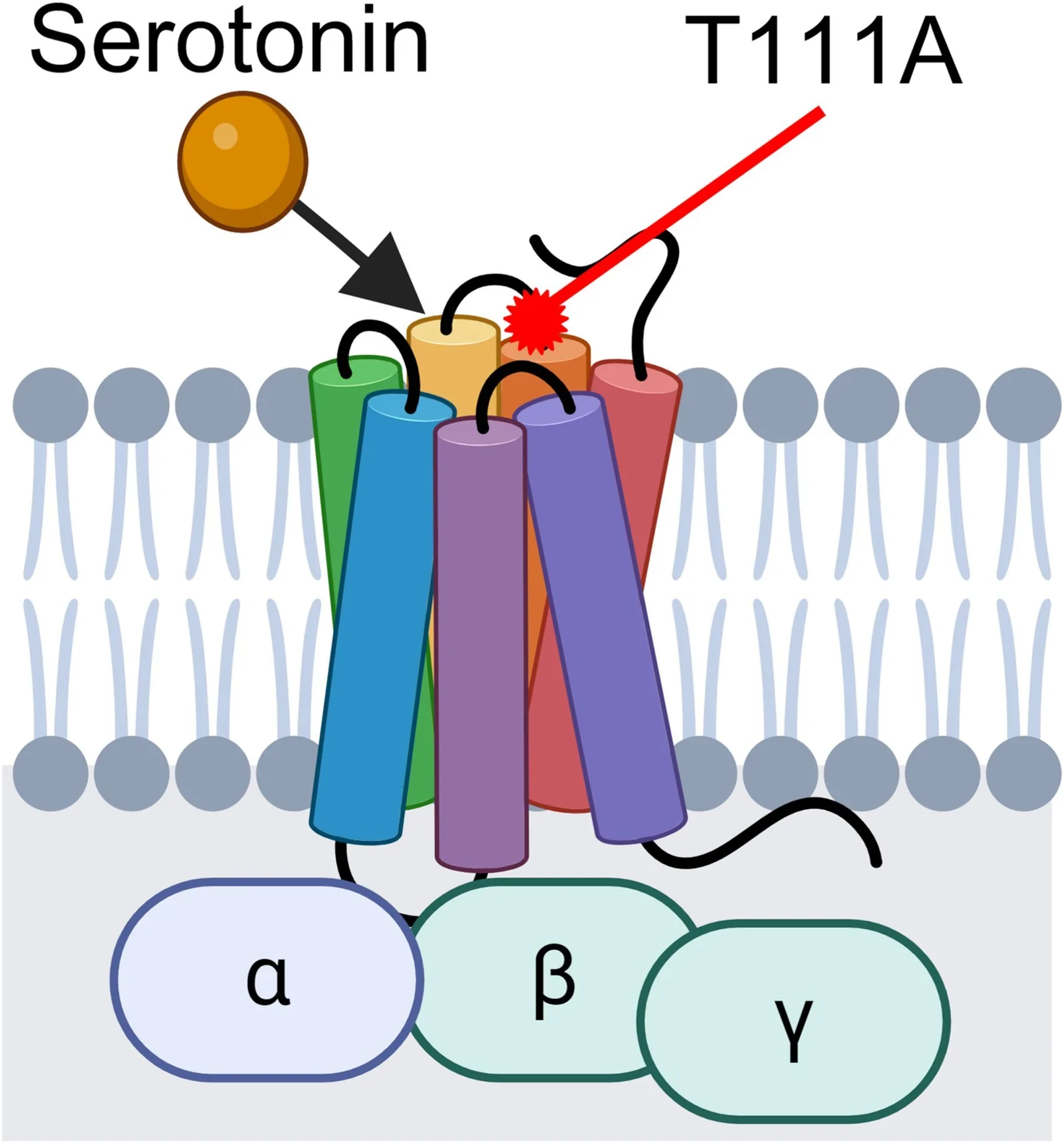
Structure of 5-HT7 in cell membrane. Location of polymorphic site in ECL1 on 5-HT7 with serotonin agonist binding facilitating interaction with heterotrimeric G-protein (α, β, γ subunits shown). Figure created in BioRender.

Two compelling candidate genes in the serotonin signaling pathway, known to directly interact and contribute to aggressive behavior, the serotonin receptor, 5-HT7 (GeneID:726702), and the guanine nucleotide-binding protein subunit gamma, Gγ (GeneID:725453) (Cifariello et al. 2008; Sarkisyan et al. 2010; Gellynck et al. 2013). 5-HT7 and Gγ are both proximal to signals of selection for European ancestry in Puerto Rican honey bees (Table 1, Fig. 3). These proteins are involved with the serotonin GPCR signaling pathways and found within the enriched GO pathways related to signaling (GO:0051716, GO:0023052, GO:0007154, GO:0007165). 5-HT7 is in the family of GPCRs that is activated by serotonin and binds with heterotrimeric G-proteins, of which Gγ is a subunit. This family of proteins plays a crucial role in neuronal morphology and has been shown to regulate aggression in Drosophila (Kvachnina et al. 2005; Dierick and Greenspan 2007). In the Asian honey bee, *Apis cerana*, 5-HT7 is seen to have been under repeated selection across different subspecies as they adapted to diverse environments (Ji et al. 2020). This repeated selection suggests that adaptations in serotonin signaling pathways could be crucial for behavioral adjustments in foraging and social organization, enabling these bees to thrive in varying ecological niches. Gγ was reported to be under selection in Puerto Rican honey bees (Avalos et al. 2017). Notably there is the non-synonymous mutation, T111A (Fig. 4) at chr1:15809746 (Table S5), found only within European honey bee parental panels that corresponds to the extracellular loop 1 (ECL1) domain, adjacent to the predicted ligand binding domain (Wheatley et al. 2012; Wang et al. 2023) which could influence the binding affinity of 5-HT7. The substitution of an Alanine, hydrophobic amino acid residue, for Threonine, a polar residue at the binding ligand binding region presents an appealing candidate for detailed biochemical investigation and further experimental phenotypic work.

There is one region, on chromosome 7, that has signals of selection for European ancestry shared among both Africanized honey bee populations in Puerto Rico and Mesoamerica: peaks PR11 and MA2 (Table 1, Fig. 3). The most proximal gene for each peak is the Forkhead Box Protein O, FOXO (GeneID:727091) which is also found in enriched GO pathways for both populations (GO:0008285, GO:0040008) (Table 3) and plays a role in overwintering (Aurori et al. 2014). The FOXO protein is a transcription factor involved in regulating cellular processes such as apoptosis, cell-cycle control, glucose metabolism, oxidative stress resistance, and is thought to play a major role in caste differentiation and division of labor by regulating insulin signaling (Ament et al. 2008; De Azevedo and Hartfelder 2008). Selection acting differently across *A. m. scutellata* populations in the region around the FOXO gene was reported in one previous study (Fuller et al. 2015). They found gene flow among populations, but a distinct loss of heterozygosity around the FOXO locus in honey bees from northern desert regions as opposed to those from more temperate savannah regions. The region around the FOXO was also shown to be differentially methylated during winter, potentially playing a role in regulation of the hypopharyngeal gland (Wang et al. 2020).

## Discussion

Genes where European ancestry is uniquely selected within the Puerto Rican population are strong candidates contributing to the evolution of docile behavior in this population. Proteins that we identified are associated with various cellular and developmental processes including cell growth, differentiation, organ morphogenesis, gene expression regulation, and cell adhesion, emphasizing the complex genetic basis underpinning the rapid adaptation of these honey bees to local environmental and human-mediated pressures. One of the most compelling of these being the 5-HT7 in Puerto Rican honey bees. 5-HT7 has been widely demonstrated in prior studies to influence behavior, and specifically aggression in other a range of species (Cifariello et al. 2008; Sarkisyan et al. 2010; Gellynck et al. 2013) and implicated in the domestication of dogs, chickens, and foxes (Trut et al. 2009; Agnvall et al. 2015; Cagan and Blass 2016). Signs of selection also occur immediately adjacent to the gene encoding the gamma subunit of the heterotrimeric G-protein that directly interacts with 5-HT7. The presence of the NMDA receptor subunit 2 and a probable ras GTPase-activating protein further implies an association with the GPCR signaling pathways in the development of this behavioral phenotype. PAX-6 and Laminin subunit alpha-1 are key regulators of neuronal development, highlighting a number of other candidates that could be further studied. An interesting candidate for adaptation for AHBs as a whole is the FOXO gene on chromosome 7, a region previously identified in Puerto Rican honey bees through a genome-wide association study that used pooled sequencing data from hives, which were assessed for their defensive response (Avalos et al. 2020). FOXO could be fundamental to bees surviving seasonal fluctuations in food availability. Worker bees surviving is a fundamental requirement for successful overwintering, which can be enhanced by reducing unnecessary metabolic activities. FOX proteins are widely known as transcription factors that play important roles in regulating the expression of genes involved in cell growth, proliferation, differentiation, and longevity (Ogg et al. 1997; Huang and Tindall 2007). FOXO is linked to juvenile hormone synthesis, impacting caste diversity in bees by affecting size, morphology, and physiology, illustrating its role in the complex social structure of bee colonies (Corona et al. 2016).

One prior study of these Puerto Rican honey bees modeled ancestry-specific selection under the assumption that soft selective sweeps led to the emergence of reduced aggression within and not necessarily due to introgression of European haplotypes from the local European honey bees (Avalos et al. 2017). This led to the detection of a number of candidate loci they further subset for regions that were also under selection during the evolution of the European honey bee. They identified haplotype blocks found in neither parent that rose to high frequency within the Puerto Rican population, with the largest regions on chromosomes 8, 9, and 15. These results have limited overlap with our study, with the only shared putative signal of selection near Gγ. Soft sweeps are an appealing potential contributor to rapid adaptation because variation is generally already present in the population. However, soft selective sweeps can be difficult to detect when relying on allele frequencies for the detection of extended haplotype homozygosity in populations with complex demographic histories, high recombination rates, continuous gene flow and high genetic diversity (Tang et al. 2007; Garud 2023; Panigrahi et al. 2023) making these methods poorly suited for Africanized honey bee populations (Avalos et al. 2017).

In comparison, AHMM-S does not rely directly on the frequencies of individual SNPs, but leverages local ancestry inference to detect regions that are inconsistent with a simple neutral model. This leads to a higher power to detect ancestry-specific selection and the fixation of ancestral haplotypes, without the reliance on the significant variation in the frequency of individual loci. Additionally, our study leveraged data from selection on European and African ancestry, as opposed to Africanized ancestry, which allows for detection of selection acting on European specific haplotypes. Leveraging the comparison to different populations of AHBs further allows us to identify regions with different ancestral profiles and under differential selection among two phenotypically distinct populations. The high selection coefficients and near fixation for these haplotypes indicate a strong selective pressure, which previous analyses and simulations have shown to be robust in cases of continuous gene flow, dominant/recessive selection, and segregation in the donor population (Svedberg et al. 2021). Our approach did not detect the same signatures of selection on chromosomes 8, 9 and 15 shown in prior studies of this population (Avalos et al. 2017). However, one gene found in each of our results, Gγ, is involved in serotonin signaling and implicated in aggression. This implies that there is limited overlap between a more typical soft-sweep and local ancestry aware analysis, highlighting the value of local ancestry aware selection scans as a complement to standard scans for natural selection.

Our study illustrates the dynamic interplay of genetic and environmental factors in shaping the behavior and genetic composition of Puerto Rican honey bee populations. Through detailed genomic analyses, we've uncovered evidence of ancestry-specific selection, where specific loci and pathways, particularly those related to neuronal development, metabolism, and behavior, have been preferentially selected. This underscores the complexity of evolutionary responses to environmental pressures and emphasizes the role of genetic admixture in facilitating rapid adaptation. It is important to note that this study does not provide definitive evidence for ancestry-specific selection i.e. that these alleles have provided adaptation to specific environments or selective regimes. While recessive deleterious variation can generate false positives for adaptive introgression in diploid systems (Setter et al. 2020; Zhang et al. 2020), this effect is expected to be substantially reduced in haplodiploid species because haploid males expose recessive alleles to selection (Zayed 2009; Miller and Sheehan 2023). Nonetheless, female-specific recessive deleterious variants may persist, and we cannot exclude the possibility that such variants could contribute to some of the observed signals. Additionally, in complex multi-locus traits such as behavior, and more explicitly aggression, it is likely that there are many polygenic and trans regulatory interactions impacting the observed phenotypic differences. Nonetheless, ancestry-aware methods for detecting selection are a potentially valuable complement to well-established scans for selection and generate a range of plausible and interesting candidates for future functional characterization.

## Materials and Methods

### Sample Origins

Genomic sequencing data for European, African, and Africanized honey bees were accessed from the NCBI Short Read Archive repository under the bioproject ID PRJNA381313 and PRJNA516678 (Table S1) (Avalos et al. 2017; Kawakami et al. 2019). The hybrid honey bees, AHBs, were sampled from two regions, Puerto Rico (PR; 30 haploid drones) and Mesoamerica (MA; 25 haploid drones) (Avalos et al. 2017). *A. m. scutellata* (AFR; 28 haploid drones) was used for the African parental panel (Kawakami et al. 2019). Haploid drones were used exclusively to avoid the need for phasing and to eliminate the uncertainty inherent in diploid genome assemblies, taking advantage of the haplodiploid system in which males carry only a single copy of each chromosome genome-wide. To account for African introgression within North American Domestic stocks from the United States (Carpenter and Harpur 2021) and C-lineage populations across Europe (Cridland et al. 2017; Utzeri et al. 2021), we used European Domestic stocks (EUR, 30 haploid drones) from Sweden and Finland which contain both C-lineage and M-lineage ancestry for our European reference panels of our primary analysis. For supplementary analyses, we included North American domestic stocks from Illinois (NAD; 30 haploid drones; Avalos et al. 2017), C-lineage individuals from France (LIG; 20 haploid drones; Wragg et al. 2016), and M-lineage individuals from Spain (MEL; 10 haploid drones; Henriques et al. 2018), detailed in Table S1 and Figs. S1 and S4.

### Variant Calling

Prior to alignment, we quality checked and trimmed raw read files with fastp version 0.23.2 (Chen et al. 2018). We aligned trimmed reads to the honey bee reference genome (RefSeq, GCF_000002195.4_Amel_4.5) with BWA MEM version 0.7.17 (Li 2013). This honey bee reference genome was chosen due to the availability of a published fine scale recombination map (Harpur et al. 2014; Corbett-Detig et al. 2015). Aligned reads were de-duplicated with samtools version 1.17 (Danecek et al. 2021). We calculated the raw variants for each de-duplicated sample using GATK version 4.4-0 HaplotypeCaller (Van der Auwera and O’Connor 2020). The variant calling process resulted in individual variant files which were subjected to joint variant calling using GATK version 4.4-0 GenomicsDBImport and followed with GenotypeGVCFs.

Initial variant calls numbered 13,900,808. Variant sites were filtered using bcftools version 1.18 (Danecek et al. 2021). We filtered sites for biallelic SNPs with minor allele frequency greater than 0.05 and further than 5 bp from indels. Unplaced scaffolds and repetitive elements were excluded from our analysis using the masked reference genome (RefSeq, GCF_000002195.4_Amel_4.5). This resulted in a VCF containing 2,905,526 SNPs spanning the 16 linkage groups. Scripts to reproduce these and the subsequent analyses are available from https://github.com/maxgenetti/AncestrySpecificSelectionAM.

### Analysis of Population Structure

We evaluated population structure among all study samples using ADMIXTURE version 1.3.0 and PCAngsd version 1.21 (Alexander et al. 2009; Meisner and Albrechtsen 2018). Using plink version 1.09, we converted the VCF into plink bed format, thinning sites to 1 per kb and genotyping frequency higher than 0.9 (Purcell et al. 2007). ADMIXTURE was run using cross-validation to confirm annotated population assignments, suitability for parental panels, and the population structure. PCAngsd was run on the vcf file to validate the approximate population structure inference using ADMIXTURE (Fig. 1, Fig. S1).

We used vcf2ahmm.py to identify ancestry informative markers (AIMs) and generate Ancestry_HMM input files (Corbett-Detig and Nielsen 2017). For the Puerto Rican and Mesoamerican populations we generated input files using European and African samples to define the parental populations. Sites are filtered for a minimum per-site allele frequency difference of 0.10 between parental panels and a minimum distance of 2.5 kb and 0.01 centiMorgans (cM) using the recombination map data from (Harpur et al. 2014) and reanalyzed and provided by (Corbett-Detig et al. 2015). We performed the following analysis with a set of 67361 AIMs.

We ran Ancestry_HMM using an admixture fraction of 0.5 in a one pulse model to estimate the ancestry proportion within the admixed population. After the first run, we accepted the mean ancestry proportion estimate obtained from the admixed samples as the admixture fraction for subsequent analysis. Further runs of the program did not affect the estimated admixture fraction. A second run using these ancestry fraction estimates was then used to estimate the generation time. To quantify uncertainty in the estimated number of generations since admixture, we ran Ancestry_HMM in 200 site windows bootstrapped 1000 times per chromosome for both populations (Fig. S5). We then compared the population structure between the prior analyses with ADMIXTURE and PCAngsd.

### Signals of Ancestry-Specific Selection

We used AHMM-S to infer ancestry-specific selection by identifying loci that have experienced positive selection following admixture (Svedberg et al. 2021). AHMM-S requires population data in the Ancestry_HMM input format (Corbett-Detig and Nielsen 2017), the time since admixture in generations (t), the size of the admixture pulse (m) and the size of the admixed population (*N*). The values of t and m were estimated using Ancestry_HMM, and as AHMM-S is not particularly sensitive to a poorly estimated value of *N* (Svedberg et al. 2021), particularly on the relatively short timescales we considered here, a fixed value of 100,000 was selected for all populations. AHMM-S was run using golden section search and the forward iteration algorithm (see Svedberg et al. 2021 for details), with a window size of the entire reference panel and set to infer selection coefficients between 0.001 and 0.5. This is an example of a command line: “ahmm-s -i input_file.txt -s sample_file.txt -*p* 1 100000 0.6 -*p* 0 100 0.4 –ne 100000 –window *p* 100 –gss 2 100000 1 0.001 0.5 –full_selection_space”. Each chromosome was run separately and we inferred natural selection acting on ancestry from either ancestral population, by swapping donor and recipient populations, and running AHMM-S for each chromosome again.

We identified genomic regions with signals of natural selection, or likelihood-ratio peaks, using scipy.signal.find_peaks with a custom peak finding criteria (Virtanen et al. 2020) (available from https://github.com/maxgenetti/AncestrySpecificSelectionAM). Specifically, we filtered peaks based on a minimum likelihood ratio and prominence. Peaks were further filtered based on minimum peak proximity of 20 AIMs (0.2 cM/100 KB) to remove putative targets of selection that are especially close together and therefore may reflect selection on a single causative allele. We applied a likelihood-ratio test, using a chi square test with 1 degree of freedom (Wilks 1938), to evaluate the evidence for natural selection at each position and corrected the *P*-value distribution for multiple comparisons using a Bonferroni correction with an alpha of 0.05 (Fig. S2).

### Neutral Admixture Simulations

While the Bonferroni-corrected test is highly conservative, we further evaluated the possibility of false positives by comparing the observed likelihood ratios to a null distribution generated from 100 neutral simulations of the Puerto Rican population using SELAM (Corbett-Detig and Jones 2016), a forward-in-time admixture simulator, as suggested by Svedberg et al. (2021). Simulations were performed under demographic parameters matching those estimated for the population in our analyses and consistent with published values (Avalos et al. 2017; Calfee et al. 2020), to capture the expected variance in ancestry tract length distributions and admixture fractions under a neutral model. Specifically, we modeled an AFR population with initial admixture pulse of 0.3 EUR ancestry, followed by 100 generations of random mating, a severe bottleneck reducing the population to four admixed individuals, representing a single mated queen, and a second pulse of 0.5 EUR ancestry which reflects the resident honey bee population in Puerto Rico. This was followed by a 50-generation bottleneck at 1,000 individuals. Simulated chromosomes were converted into Ancestry_HMM input files and processed with Ancestry_HMM and AHMM-S to generate a null distribution of log-likelihood ratios under neutrality. Likelihood-ratio peaks in the simulated data were identified using scipy.signal.find_peaks (Virtanen et al. 2020) with the same peak-finding criteria as for the real data (available from https://github.com/maxgenetti/AncestrySpecificSelectionAM), resulting in a mean of 0.67 and 0.51 expected false discoveries of ancestry-specific selected sites per genome for AFR and EUR (Fig. S6). While it is unlikely that the full demographic history of the Puerto Rican population can be precisely inferred or modeled, our neutral simulations were parameterized to match the major known events and deliberately used conservative values in order to minimize the possibility of false discovery of ancestry-specific selection. These values are below the threshold of one false discovery per genome, as recommended in the AHMM-S methods (Svedberg et al. 2021).

To demonstrate that signals of selection are independent of extended haplotype homozygosity or other underlying genomic features, we calculated the nucleotide diversity (*π*), exon density, and recombination rate in 100KB windows around each AIM (Fig. S3). Following standard practices (Calfee et al. 2021, Langdon et al. 2024), we evaluated correlations between selection signals and genomic features using Spearman's rank correlation on data binned into deciles. (Table S3). To further validate the selection of AIMs, we calculated Pearson's correlation coefficient and the *P*-value between the Puerto Rican and Mesoamerican likelihood ratio of selection on European and African ancestry.

### Evaluating A Population Bottleneck in Puerto Rico

To evaluate the potential impact of a population bottleneck causing a loss of genetic diversity, we measure African and European haplotype diversity within the Puerto Rican and Mesoamerican populations. Ancestry tracts for each sample are identified using the Viterbi decoding option of Ancestry_HMM. We sample individuals from each population for each inferred ancestry in 200 bp windows. When the number of individuals is not equivalent, the more abundant is randomly down-sampled. The number of unique haplotypes for each window are counted using biallelic SNPs from the filtered VCF. We then compare haplotype diversity for each ancestry between the Puerto Rican and Mesoamerican populations (Table 2).

### Genes of Interest and GO Enrichment Analysis

We recorded protein coding genes proximal to and within 50 kb of the peaks that remained significant after multiple testing selection criteria (Table 1, Table S2). We used GO annotations from the Hymenoptera Genome Database to perform enrichment analyses focused on both Biological Process categories to identify enriched GO terms related to European and African specific selection separately (Walsh et al. 2022). To mitigate potential bias from genes in proximity sharing annotations, we only use the most proximal genes for GO-enrichment analysis and account for bias from gene length through a permutation procedure that retains the lengths of coding elements (Kofler and Schlötterer 2012). In short, we randomly sample peaks from AIMs in non-overlapping genomic regions, consistent with our peak filtering criteria, 10 million times to produce GO term distributions for each ancestry-specific selection scenario. We use these distributions to calculate *P*-values for GO terms with at least 2 observations. We apply the Benjamini-Hochberg Procedure for multiple testing correction, setting a False Discovery Rate (FDR) cut-off at 0.05, to identify statistically significant GO terms and the associated genes (Table 3).

## Supplementary Material

evaf217_Supplementary_Data

## Data Availability

A full copy of the code used for variant calling, custom Ancestry_HMM and AHMM-S pipeline, SELAM simulations, and calculating population genetic summary statistics in haploid species are available on github (https://github.com/maxgenetti/AncestrySpecificSelectionAM).
