# Correction to: Signals of Ancestry-Specific Selection in Gentle Africanized Honey Bees

**DOI:** 10.1093/gbe/evag250

**Published:** 2026-10-08

This is a correction to: Maximilian Genetti, Russell Corbett-Detig, Signals of Ancestry-Specific Selection in Gentle Africanized Honey Bees, *Genome Biology and Evolution*, Volume 17, Issue 12, December 2025, evaf217, https://doi.org/10.1093/gbe/evaf217

In the originally published version of this manuscript, a 'Conflict of Interest' statement was omitted.

The following declaration has been added to the paper:

"R.C.-D. reports consulting for International Responder Systems. This consulting relationship had no role in the design, conduct, interpretation, or publication of this study.The authors declare no other competing interests".

