# Supplementary material for: Signals of Ancestry-Specific Selection in Gentle Africanized Honey Bees
Source: Genome Biol Evol. 2025 Nov 18;17(12):evaf217. doi: 10.1093/gbe/evaf217 (PMC12673328; doi:10.1093/gbe/evaf217)
Supplement: evaf217_Supplementary_Data [file evaf217_supplementary_data.pdf]

Supporting Information

Table S1. Genomic sequencing data.

BioProject	Sample Name	Population
PRJNA516678	FB_B12_3_bond	EUR
PRJNA516678	FB_B12_5_bond	EUR
PRJNA516678	FB_B12_9_bond	EUR
PRJNA516678	GF2_1_al	EUR
PRJNA516678	GF2_6_al	EUR
PRJNA516678	GF2_9_al	EUR
PRJNA516678	K5_2_al	EUR
PRJNA516678	K5_3_al	EUR
PRJNA516678	K5_6_al	EUR
PRJNA516678	KL1_2_bond	EUR
PRJNA516678	KL1_3_bond	EUR
PRJNA516678	KL1_6_bond	EUR
PRJNA516678	N1_1_al	EUR
PRJNA516678	N1_4_al	EUR
PRJNA516678	N1_5_al	EUR
PRJNA516678	NB1_2_al	EUR
PRJNA516678	NB1_3_al	EUR
PRJNA516678	NB1_7_al	EUR
PRJNA516678	NW1_2_bond	EUR
PRJNA516678	NW1_5_bond	EUR
PRJNA516678	NW1_6_bond	EUR
PRJNA516678	T3_1_al	EUR
PRJNA516678	T3_5_al	EUR
PRJNA516678	T3_6_al	EUR
PRJNA516678	TB1_1_al	EUR
PRJNA516678	TB1_3_al	EUR
PRJNA516678	TB1_5_al	EUR
PRJNA516678	TC2_1_al	EUR
PRJNA516678	TC2_4_al	EUR
PRJNA516678	TC2_5_al	EUR
PRJNA516678	scu_d_1_1	AFR
PRJNA516678	scu_d_1_10	AFR
PRJNA516678	scu_d_1_3	AFR
PRJNA516678	scu_d_1_4	AFR
PRJNA516678	scu_d_1_5	AFR
PRJNA516678	scu_d_1_6	AFR

PRJNA516678	scu_d_1_7	AFR
PRJNA516678	scu_d_1_8	AFR
PRJNA516678	scu_d_1_9	AFR
PRJNA516678	scu_d_2_1	AFR
PRJNA516678	scu_d_2_10	AFR
PRJNA516678	scu_d_2_2	AFR
PRJNA516678	scu_d_2_3	AFR
PRJNA516678	scu_d_2_4	AFR
PRJNA516678	scu_d_2_5	AFR
PRJNA516678	scu_d_2_6	AFR
PRJNA516678	scu_d_2_7	AFR
PRJNA516678	scu_d_2_8	AFR
PRJNA516678	scu_d_2_9	AFR
PRJNA516678	scu_lt_5_1	AFR
PRJNA516678	scu_lt_5_2	AFR
PRJNA516678	scu_lt_5_3	AFR
PRJNA516678	scu_lt_5_4	AFR
PRJNA516678	scu_lt_5_5	AFR
PRJNA516678	scu_lt_5_6	AFR
PRJNA516678	scu_lt_5_7	AFR
PRJNA516678	scu_lt_5_8	AFR
PRJNA516678	scu_lt_5_9	AFR
PRJNA381313	b2M26_RSZAIPI005065-37_AHB	MA
PRJNA381313	b2M28_RSZAIPI005066-39_AHB	MA
PRJNA381313	b2M31_RSZAIPI005404-140_AHB	MA
PRJNA381313	b2M33_RSZAIPI005490-141_AHB	MA
PRJNA381313	b2M34_RSZAIPI005491-142_AHB	MA
PRJNA381313	b2M48_RSZAIPI005494-146_AHB	MA
PRJNA381313	b2C1A_RSZAIPI005495-147_AHB	MA
PRJNA381313	b2C2A_RSZAIPI005496-148_AHB	MA
PRJNA381313	b2C5A_RSZAIPI005497-149_AHB	MA
PRJNA381313	b2C7A_RSZAIPI005498-150_AHB	MA
PRJNA381313	b2C8A_RSZAIPI005499-152_AHB	MA
PRJNA381313	b2C9A_RSZAIPI005500-153_AHB	MA
PRJNA381313	b2C10A_RSZAIPI005501-155_AHB	MA
PRJNA381313	b2C11A_RSZAIPI005502-159_AHB	MA
PRJNA381313	b2C12A_RSZAIPI005503-160_AHB	MA
PRJNA381313	b2C16A_RSZAIPI005504-161_AHB	MA
PRJNA381313	b2C17A_RSZAIPI005505-162_AHB	MA
PRJNA381313	b2C20A_RSZAIPI005506-167_AHB	MA

PRJNA381313	b2C23A_RSZAIPI005507-169_AHB	MA
PRJNA381313	b2C25A_RSZAIPI005508-170_AHB	MA
PRJNA381313	115MEX_WHAIPI005599-64_AHB	MA
PRJNA381313	110MEX_WHAIPI005600-66_AHB	MA
PRJNA381313	52MEX_WHAIPI005627-71_AHB	MA
PRJNA381313	33MEX_WHAIPI005628-72_AHB	MA
PRJNA381313	26MEX_WHAIPI005629-73_AHB	MA
PRJNA381313	44PR_WHAIPI005639-95_gAHB	PR
PRJNA381313	32PR_WHAIPI005637-92_gAHB	PR
PRJNA381313	33PR_WHAIPI005638-93_gAHB	PR
PRJNA381313	41PR_WHAIPI005640-97_gAHB	PR
PRJNA381313	1PR_WHAIPI005630-74_gAHB	PR
PRJNA381313	2PR_WHAIPI005631-75_gAHB	PR
PRJNA381313	7PR_WHAIPI005632-79_gAHB	PR
PRJNA381313	11PR_WHAIPI005633-80_gAHB	PR
PRJNA381313	12PR_WHAIPI005634-81_gAHB	PR
PRJNA381313	20PR_WHAIPI005635-85_gAHB	PR
PRJNA381313	31PR_WHAIPI005636-90_gAHB	PR
PRJNA381313	b21GPR_RSZAIPI005061-32_gAHB	PR
PRJNA381313	b22GPR_RSZAIPI005062-33_gAHB	PR
PRJNA381313	b219CAPR_RSZAIPI005063-34_gAHB	PR
PRJNA381313	b238_RSZAIPI005400-135_gAHB	PR
PRJNA381313	b225_RSZAIPI005403-132_gAHB	PR
PRJNA381313	b218_RSZAIPI005406-130_gAHB	PR
PRJNA381313	b228_RSZAIPI005411-133_gAHB	PR
PRJNA381313	b29_RSZAIPI005414-125_gAHB	PR
PRJNA381313	b219_RSZAIPI005415-131_gAHB	PR
PRJNA381313	b214_RSZAIPI005418-129_gAHB	PR
PRJNA381313	b24GPR_RSZAIPI005410-111_gAHB	PR
PRJNA381313	b26GPR_RSZAIPI005412-113_gAHB	PR
PRJNA381313	b221CAPR_RSZAIPI005413-119_gAHB	PR
PRJNA381313	b28_RSZAIPI005416-123_gAHB	PR
PRJNA381313	b2BA4PR_RSZAIPI005417-122_gAHB	PR
PRJNA381313	b220CAPR_RSZAIPI005419-117_gAHB	PR
PRJNA381313	b29CPR_RSZAIPI005420-114_gAHB	PR
PRJNA381313	b212ABPR_RSZAIPI005425-115_gAHB	PR
PRJNA381313	b214ABPR_RSZAIPI005427-116_gAHB	PR
PRJNA381313	B08_WHAIPI005557-26_EHB	NAD
PRJNA381313	B10A_WHAIPI005562-27_EHB	NAD
PRJNA381313	b2B14-2_RSZAIPI005422-92_EHB	NAD

PRJNA381313	b2W144_RSZAIPI005409-93_EHB	NAD
PRJNA381313	b2W172_RSZAIPI005398-94_EHB	NAD
PRJNA381313	b2W187-1_RSZAIPI005060-31_EHB	NAD
PRJNA381313	b2W190_RSZAIPI005405-95_EHB	NAD
PRJNA381313	b2W193_RSZAIPI005408-96_EHB	NAD
PRJNA381313	b2W198_RSZAIPI005421-97_EHB	NAD
PRJNA381313	b2Y10-2_RSZAIPI005407-102_EHB	NAD
PRJNA381313	b2Y13-2_RSZAIPI005424-106_EHB	NAD
PRJNA381313	b2Y19-3_RSZAIPI005426-107_EHB	NAD
PRJNA381313	b2Y25-2_RSZAIPI005402-108_EHB	NAD
PRJNA381313	b2Y41-1_RSZAIPI005399-109_EHB	NAD
PRJNA381313	b2Y6-1_RSZAIPI005423-98_EHB	NAD
PRJNA381313	W153-40_WHAIPI005572-43_EHB	NAD
PRJNA381313	W174_WHAIPI005561-14_EHB	NAD
PRJNA381313	W177_WHAIPI005565-15_EHB	NAD
PRJNA381313	W180_WHAIPI005551-16_EHB	NAD
PRJNA381313	W189_WHAIPI005552-17_EHB	NAD
PRJNA381313	W197_WHAIPI005553-18_EHB	NAD
PRJNA381314	W199_WHAIPI005554-19_EHB	NAD
PRJNA381315	W200_WHAIPI005563-20_EHB	NAD
PRJNA381316	W22_WHAIPI005556-13_EHB	NAD
PRJNA381317	Y2_WHAIPI005560-21_EHB	NAD
PRJNA381318	Y4_WHAIPI005555-22_EHB	NAD
PRJNA381319	Y4-39_WHAIPI005571-42_EHB	NAD
PRJNA381320	Y5_WHAIPI005558-23_EHB	NAD
PRJNA381321	Y7_WHAIPI005549-24_EHB	NAD
PRJNA381322	Y9_WHAIPI005564-25_EHB	NAD
PRJNA893245	2022	M
PRJNA893245	2068	M
PRJNA893245	2106	M
PRJNA893245	2160	M
PRJNA893245	2242	M
PRJNA893245	2284	M
PRJNA893245	2368	M
PRJNA893245	2485	M
PRJNA893245	2549	M
PRJNA893245	2652	M
PRJNA311274	ITA10A	C
PRJNA311274	ITA12A	C
PRJNA311274	ITA14A	C

PRJNA311274	ITA16A	C
PRJNA311274	ITA18A	C
PRJNA311274	ITA20A	C
PRJNA311274	ITA2A	C
PRJNA311274	ITA4A	C
PRJNA311274	ITA6A	C
PRJNA311274	ITA8A	C
PRJNA311274	BE10	C
PRJNA311274	BE2	C
PRJNA311274	BE4	C
PRJNA311274	BE6	C
PRJNA311274	BE8	C
PRJNA311274	SL1	C
PRJNA311274	SL3	C
PRJNA311274	SL5	C
PRJNA311274	SL7	C
PRJNA311274	SL9	C

Table S1. Genomic sequencing data. Bioproject, sample name, and population for European, African, and Africanized honey bees were accessed from the NCBI Short Read Archive repository.

Table S2. Log-Likelihood-Ratios.

Pop	Anc	Peak	GeneID	Alt ID	Chr	Position	Distance (bp)	Selection Coefficient	Log-likelihood Ratio
PR	AFR	PF1	726255	GB48631	5	7767124	39983	0.05777	25.578
PR	AFR	PF1	412965	GB48632	5	7767124	43464	0.05777	25.578
PR	AFR	PF1	409698	GB48660	5	7767124	40818	0.05777	25.578
PR	AFR	PF1	413542	GB48661	5	7767124	35511	0.05777	25.578
PR	AFR	PF1	726309	GB48664	5	7767124	26421	0.05777	25.578
PR	AFR	PF1	409818	GB48665	5	7767124	0	0.05777	25.578
PR	AFR	PF3	410229	GB17781	10	6057267	-36185	0.07646	32.464
PR	AFR	PF4	551706	GB54226	10	8141101	44833	0.13892	45.664
PR	AFR	PF4	724333	GB54295	10	8141101	0	0.13892	45.664
PR	AFR	PF5	725924	GB42096	11	4256369	0	0.05541	31.638
PR	AFR	PF7	552313	GB49826	11	6101187	26084	0.05403	29.921
PR	AFR	PF7	410118	GB49827	11	6101187	-7184	0.05403	29.921
PR	AFR	PF7	552308	GB49828	11	6101187	-12524	0.05403	29.921
PR	AFR	PF7	724953	GB49829	11	6101187	-14862	0.05403	29.921
PR	AFR	PF7	725084	GB49830	11	6101187	-38942	0.05403	29.921
PR	AFR	PF7	412972	GB49837	11	6101187	-42165	0.05403	29.921
PR	AFR	PF7	725050	GB49838	11	6101187	-36388	0.05403	29.921
PR	AFR	PF7	412842	GB49839	11	6101187	-17930	0.05403	29.921
PR	AFR	PF7	552367	GB49843	11	6101187	2197	0.05403	29.921
PR	AFR	PF7	412422	GB49844	11	6101187	14174	0.05403	29.921
PR	AFR	PF7	412215	GB49845	11	6101187	42295	0.05403	29.921
PR	AFR	PF7	102654083		11	6101187	-1894	0.05403	29.921
PR	AFR	PF7	412247		11	6101187	-45435	0.05403	29.921
PR	AFR	PF7	100577455		11	6101187	-46289	0.05403	29.921
PR	EUR	PR1	100578309	GB50339	1	2929896	-48769	0.10692	25.687
PR	EUR	PR1	724238	GB50342	1	2929896	31864	0.10692	25.687
PR	EUR	PR1	724331	GB50364	1	2929896	-33497	0.10692	25.687
PR	EUR	PR1	724375	GB50365	1	2929896	-37048	0.10692	25.687
PR	EUR	PR1	551450	GB50366	1	2929896	-45956	0.10692	25.687
PR	EUR	PR2	724117	GB53885	1	15255347	22251	0.10675	40.300
PR	EUR	PR2	100578389	GB53886	1	15255347	-43549	0.10675	40.300
PR	EUR	PR2	100578347	GB53888	1	15255347	19428	0.10675	40.300
PR	EUR	PR2	107963975		1	15255347	10434	0.10675	40.300
PR	EUR	PR2	102654405		1	15255347	14865	0.10675	40.300
PR	EUR	PR2	102654781		1	15255347	28973	0.10675	40.300

PR	EUR	PR3	726702	GB14021	1	15851999	0	0.07849	25.317
PR	EUR	PR4	552032	GB47419	1	17483461	-47318	0.1063	24.732
PR	EUR	PR4	413650	GB47420	1	17483461	41912	0.1063	24.732
PR	EUR	PR4	725946	GB47468	1	17483461	47150	0.1063	24.732
PR	EUR	PR4	413649	GB47469	1	17483461	39829	0.1063	24.732
PR	EUR	PR4	410009	GB47470	1	17483461	0	0.1063	24.732
PR	EUR	PR4	100576231		1	17483461	-40085	0.1063	24.732
PR	EUR	PR5	726766	GB48923	1	22337326	0	0.10675	43.008
PR	EUR	PR5	725976	GB48924	1	22337326	-8563	0.10675	43.008
PR	EUR	PR5	551637	GB48925	1	22337326	-20927	0.10675	43.008
PR	EUR	PR5	409225	GB48926	1	22337326	-29453	0.10675	43.008
PR	EUR	PR5	726708	GB48927	1	22337326	-38751	0.10675	43.008
PR	EUR	PR5	414042	GB48928	1	22337326	-49499	0.10675	43.008
PR	EUR	PR5	726647	GB48946	1	22337326	-44930	0.10675	43.008
PR	EUR	PR5	100576242	GB48948	1	22337326	-41180	0.10675	43.008
PR	EUR	PR5	551725	GB48949	1	22337326	-32089	0.10675	43.008
PR	EUR	PR5	409226	GB48951	1	22337326	-25109	0.10675	43.008
PR	EUR	PR5	412412	GB48955	1	22337326	-3954	0.10675	43.008
PR	EUR	PR5	726777	GB48956	1	22337326	6441	0.10675	43.008
PR	EUR	PR5	107966053		1	22337326	-11584	0.10675	43.008
PR	EUR	PR6	100578543	GB53930	1	27886171	37620	0.0819	26.215
PR	EUR	PR6	410775	GB53931	1	27886171	0	0.0819	26.215
PR	EUR	PR7	100578771	GB47036	3	10566232	0	0.10675	26.810
PR	EUR	PR8	408779	GB44091	4	9377455	13110	0.08595	28.636
PR	EUR	PR8	413423	GB44117	4	9377455	-138	0.08595	28.636
PR	EUR	PR8	726681	GB44119	4	9377455	2388	0.08595	28.636
PR	EUR	PR9	409115	GB44059	4	9749904	41489	0.08493	28.108
PR	EUR	PR9	100578015	GB44060	4	9749904	35095	0.08493	28.108
PR	EUR	PR9	409117	GB44061	4	9749904	19712	0.08493	28.108
PR	EUR	PR9	411582	GB44062	4	9749904	11082	0.08493	28.108
PR	EUR	PR9	725678	GB44063	4	9749904	-9076	0.08493	28.108
PR	EUR	PR9	725703	GB44064	4	9749904	-12712	0.08493	28.108
PR	EUR	PR9	552792	GB44065	4	9749904	-22541	0.08493	28.108
PR	EUR	PR9	413138	GB44067	4	9749904	-28882	0.08493	28.108
PR	EUR	PR9	725897	GB44069	4	9749904	-39019	0.08493	28.108
PR	EUR	PR9	413141	GB44143	4	9749904	-41949	0.08493	28.108
PR	EUR	PR9	413139	GB44144	4	9749904	-35383	0.08493	28.108
PR	EUR	PR9	409789	GB44146	4	9749904	-14643	0.08493	28.108

PR	EUR	PR9	413137	GB44147	4	9749904	-10299	0.08493	28.108
PR	EUR	PR9	409788	GB44148	4	9749904	-1978	0.08493	28.108
PR	EUR	PR9	725627	GB44149	4	9749904	0	0.08493	28.108
PR	EUR	PR9	409118	GB44152	4	9749904	6055	0.08493	28.108
PR	EUR	PR9	552809	GB44153	4	9749904	16635	0.08493	28.108
PR	EUR	PR9	411581	GB44154	4	9749904	17551	0.08493	28.108
PR	EUR	PR9	725434	GB44155	4	9749904	24704	0.08493	28.108
PR	EUR	PR9	411579	GB44156	4	9749904	31594	0.08493	28.108
PR	EUR	PR9	408786	GB44159	4	9749904	43342	0.08493	28.108
PR	EUR	PR9	102655905		4	9749904	4714	0.08493	28.108
PR	EUR	PR9	102655592		4	9749904	-27771	0.08493	28.108
PR	EUR	PR9	102655452		4	9749904	-38264	0.08493	28.108
PR	EUR	PR11	727091	GB48301	7	6915854	0	0.0896	30.586
PR	EUR	PR12	412818	GB48097	7	12200935	0	0.10636	26.820
PR	EUR	PR12	100577159	GB48183	7	12200935	0	0.10636	26.820
PR	EUR	PR13	725453	GB10017	14	1847557	-249	0.08005	25.987
PR	EUR	PR13	725620	GB16493	14	1847557	2235	0.08005	25.987
PR	EUR	PR13	100576136	GB48468	14	1847557	22535	0.08005	25.987
PR	EUR	PR13	413623	GB48469	14	1847557	10008	0.08005	25.987
PR	EUR	PR13	413481	GB48474	14	1847557	-22826	0.08005	25.987
PR	EUR	PR14	726094	GB50573	15	4658368	-47346	0.08509	24.860
PR	EUR	PR14	726125	GB50575	15	4658368	-38708	0.08509	24.860
PR	EUR	PR14	726155	GB50577	15	4658368	-20090	0.08509	24.860
PR	EUR	PR14	726185	GB50578	15	4658368	-18281	0.08509	24.860
PR	EUR	PR14	726206	GB50579	15	4658368	-15203	0.08509	24.860
PR	EUR	PR14	726229	GB50580	15	4658368	-11886	0.08509	24.860
PR	EUR	PR14	102656231		15	4658368	-40710	0.08509	24.860
MA	AFR	MF1	408958	GB40523	8	3246532	0	0.06444	25.907
MA	AFR	MF2	413200	GB46165	15	1462405	45324	0.05599	31.092
MA	AFR	MF2	725717	GB46167	15	1462405	39485	0.05599	31.092
MA	AFR	MF2	551984	GB46169	15	1462405	31880	0.05599	31.092
MA	AFR	MF2	100577418	GB46170	15	1462405	-13303	0.05599	31.092
MA	AFR	MF2	726566	GB46173	15	1462405	-39993	0.05599	31.092
MA	AFR	MF2	100578894	GB46174	15	1462405	-44614	0.05599	31.092
MA	AFR	MF2	412430	GB46177	15	1462405	-48943	0.05599	31.092
MA	AFR	MF2	726601	GB46218	15	1462405	-44410	0.05599	31.092
MA	AFR	MF2	726528	GB46219	15	1462405	-26600	0.05599	31.092
MA	AFR	MF2	725811	GB46220	15	1462405	-23901	0.05599	31.092

MA	AFR	MF2	552668	GB46221	15	1462405	-16912	0.05599	31.092
MA	AFR	MF2	677674	GB46222	15	1462405	-7266	0.05599	31.092
MA	AFR	MF2	677673	GB46223	15	1462405	-1318	0.05599	31.092
MA	AFR	MF2	551719	GB46225	15	1462405	4837	0.05599	31.092
MA	AFR	MF2	552478	GB46226	15	1462405	9278	0.05599	31.092
MA	AFR	MF2	677677	GB46227	15	1462405	13716	0.05599	31.092
MA	AFR	MF2	551935	GB46230	15	1462405	23942	0.05599	31.092
MA	AFR	MF2	726435	GB46231	15	1462405	28799	0.05599	31.092
MA	EUR	MR1	406073	GB55032	5	3036328	0	0.07259	28.305
MA	EUR	MR2	552152	GB48293	7	6744151	-7329	0.07295	59.833
MA	EUR	MR2	412552	GB48300	7	6744151	-14863	0.07295	59.833
MA	EUR	MR2	727091	GB48301	7	6744151	6644	0.07295	59.833
MA	EUR	MR2	102654121		7	6744151	-19385	0.07295	59.833
MA	EUR	MR3	724773	GB49862	13	8690069	-3335	0.05259	26.640
MA	EUR	MR3	102656452		13	8690069	-36381	0.05259	26.640
MA	EUR	MR4	725053	GB12518	13	8925953	0	0.06204	26.769
MA	EUR	MR4	725111	GB49909	13	8925953	43151	0.06204	26.769
MA	EUR	MR4	408438	GB49911	13	8925953	30277	0.06204	26.769

Table S2. Log-Likelihood-Ratios. The log-likelihood ratios and genes within 50 KB of peaks identified using scipy.signal.find_peaks.

Table S3. Spearman's rank correlation coefficient (ρ) between Signals of Selection and Nucleotide Diversity, Exon Density and Recombination Rate.

Population	Ancestry	Feature	Spearman's ρ	p-value
PR	AFR	nucleotide diversity	2.61E-01	4.67E-01
PR	EUR	nucleotide diversity	-1.52E-01	6.76E-01
MA	AFR	nucleotide diversity	-6.61E-01	3.76E-02
MA	EUR	nucleotide diversity	5.52E-01	9.84E-02
PR	AFR	exon density	-9.09E-02	8.03E-01
PR	EUR	exon density	-5.45E-02	8.81E-01
MA	AFR	exon density	8.30E-01	2.94E-03
MA	EUR	exon density	-8.79E-01	8.14E-04
PR	AFR	recombination rate	-5.39E-01	1.08E-01
PR	EUR	recombination rate	6.12E-01	6.00E-02
MA	AFR	recombination rate	-6.67E-02	8.55E-01
MA	EUR	recombination rate	7.70E-01	9.22E-03

Table S3. Spearman's rank correlation coefficient (ρ) between Signals of Selection and Nucleotide Diversity, Exon Density and Recombination Rate. Correlations for the log-likelihood ratios for selection on African-like (AFR) and European-like (EUR) ancestry with nucleotide diversity, exon density, and recombination rate are reported for Puerto Rican (PR) and Mesoamerican (MA) honey bees. Nucleotide diversity, exon density, and recombination rate were calculated in 100KB windows around each Ancestry Informative Marker. All ρ values and associated p-values were calculated on data binned into deciles.

Table S4. Genes Consistent with Natural Selection.

Peak	GeneID	Description	Phenotype Association
PR1	724238	paired box protein Pax-6	Transcription factor which regulates eye formation during embryonic development with broader impacts on the formation of the central nervous system including glutamatergic neuronal differentiation (Georgala et al., 2011; Kim et al., 2014).
PR3	726702	serotonin receptor 7	In the Asian honey bee, *A. cerana*, 5-HT7 is seen to have been under repeated selection across different subspecies as they adapted to diverse environments (Ji et al., 2020).
PR4	410009	cadherin-related tumor suppressor	Upregulated in honey bees after being exposed to neuropeptides to increase their aggressivity (Paula et al., 2024).
PR5	726766	endoribonuclease Dicer	Upregulated following *Varroa* Mite infection (Zhao et al., 2019).
PR6	410775	laminin subunit alpha-1	Part of the laminin molecule, essential for cell adhesion, differentiation, migration, with mutations leading to changes in glial cell morphology (Petley-Ragan et al., 2016; Pozzi et al., 2017).
PR7	100578771	probable Ras GTPase-activating protein	Increases the rates at which heterotrimeric G protein subunits hydrolyze bound GTP (Ross & Wilkie, 2000).
PR8	413423	hemicentin-2	Hemicentin-2 has been implicated in the response to *Varroa destructor* and fungicide exposure in *A. mellifera* (Wu et al., 2023).
PR9	725627	clotting factor B	Part of the innate immune system. The putative key clotting factor (Tg) which facilitates the crosslinking of clotting factors is found in the larva of *Varroa* resistant *A. mellifera* colonies (Parker et al., 2012)
PR11	727091	forkhead box protein O	Selection acting differently across *A. m. scutellata* populations in the region around the FOXO gene was reported in one previous study linked to local ecology (Fuller et al., 2015). The region around the FOXO was also shown to be differentially methylated during winter, potentially playing a role in regulation of the Hypopharyngeal Gland in *A. mellifera* (Wang et al., 2020).
PR12	412818	NMDA receptor 2	Glutamate GPCR that plays a critical role in synaptic plasticity and memory function with potential impacts on aggression in *A. mellifera* (K. Kim et al., 2018). NMDA inhibition and excitation has been studied in *A. mellifera* for its role in learning and memory (Maleszka et al., 2000; Si et al., 2004).
PR13	725453	guanine nucleotide-binding protein subunit gamma-e	G protein subunit that is part of the GPCR signaling pathway and was reported to be under selection in Puerto Rican honey bees (Avalos et al., 2017).
PR14	726229	Osi20	Part of the syntenic coregulated Osiris gene family. Osiris genes are expressed in the developing brains of both castes and are upregulated in queens' brains of *A. mellifera* (Vieira et al., 2021).

Table S4. Genes Consistent with Natural Selection. Descriptions of the genes proximal to log-likelihood ratio peaks that are consistent with the observed Puerto Rican honey bee phenotype.

Table S5. Differentiated SNPs in Protein Coding Regions.

CHR	Position	REF	ALT	EUR REF_AF	AFR REF_AF	PR REF_AF	MA REF_AF
1	15266316	A	G	0.428571429	0	0.366666667	0.32
1	15266376	G	A	0.4	0.821428571	0.4	0.52
1	15266383	G	A	0.466666667	1	0.4	0.64
1	15266514	A	G	0.62962963	0	0.633333333	0.36
1	15266636	A	G	0.76	0	0.633333333	0.56
1	15266639	T	A	0.76	0	0.633333333	0.56
1	15806989	G	A	0.444444444	1	0.466666667	0.72
1	15809732	G	A	0.466666667	1	0.466666667	0.92
1	15809746	T	C	0.433333333	0	0.3	0.24
1	15809903	A	G	0.482758621	1	0.466666667	0.72
1	17445302	A	G	0.344827586	0	0.4	0.12
1	17445494	G	A	0.535714286	1	0.733333333	0.8
1	17445740	A	G	1	0.428571429	0.933333333	0.72
1	17445743	C	T	1	0.428571429	0.933333333	0.72
1	17446036	G	T	1	0.142857143	1	0.68
1	17446166	A	C	1	0.357142857	0.933333333	0.92
1	17446256	C	A	0.862068966	0.428571429	0.766666667	0.68
1	17446343	A	G	0.928571429	0.571428571	0.933333333	0.64
1	17446718	A	C	1	0.642857143	1	0.96
1	17446952	A	G	0.966666667	0.392857143	0.9	0.76
1	17447299	C	T	1	0.428571429	0.933333333	0.88
1	17447491	G	A	1	0.571428571	0.933333333	0.88
1	17447563	G	A	1	0.642857143	0.933333333	0.88
1	17449304	T	C	0.866666667	0.25	0.9	0.56
1	17450141	G	A	0.620689655	0.25	0.9	0.68
1	17452006	T	G	1	0.392857143	0.9	0.72
1	17452057	G	A	1	0.571428571	1	0.92
1	17452168	G	C	0.428571429	0	0.233333333	0.24
1	17452420	G	A	0.733333333	0	0.866666667	0.52
1	17454451	C	G	1	0.571428571	1	0.96
1	17456229	C	G	1	0.535714286	1	0.56
1	17456827	A	G	1	0.571428571	1	0.76
1	17457163	A	G	0.333333333	0	0.233333333	0.2
1	17459813	T	C	0.857142857	0.392857143	0.633333333	0.8
1	17459855	A	G	0.814814815	0.142857143	0.633333333	0.76
1	17462310	C	G	1	0.5	1	0.84
1	17514677	G	C	1	0.142857143	0.866666667	0.6
1	17515315	A	G	0.961538462	0.285714286	0.766666667	0.56
1	17515355	C	T	0.814814815	0.214285714	0.8	0.84
1	17516348	G	A	0.892857143	0.142857143	0.6	0.52
1	17516451	T	C	1	0.357142857	1	0.84
1	17516906	G	C	1	0.25	1	0.96
1	17517698	A	G	1	0.5	0.866666667	0.8
1	17517893	G	A	1	0.642857143	1	1
1	17517941	G	T	1	0.178571429	0.733333333	0.76
1	17518382	T	C	0.740740741	0	0.466666667	0.28
1	17518475	A	C	0.964285714	0.464285714	0.8	0.84

1	22338150	A	G	0.8	0	0.866666667	0.32
1	22339870	C	T	1	0.285714286	1	0.818181818
1	27889769	A	T	0.586206897	0	0.3	0.2
1	27897990	T	C	0.733333333	0	0.533333333	0.4
1	27899691	A	G	0.466666667	0	0.333333333	0.24
1	27901270	A	G	0.633333333	0.214285714	0.533333333	0.16
1	27901630	T	C	1	0.464285714	0.966666667	0.84
1	27902547	C	A	1	0.333333333	0.866666667	0.36
1	27903949	C	G	0.535714286	1	0.6	0.52
1	27979010	T	C	0.222222222	0.75	0.2	0.32
1	27981903	G	A	0.888888889	0	0.466666667	0.2
1	27982892	C	T	0.6	0	0.433333333	0.4
1	27983089	C	T	0.448275862	0.857142857	0.5	0.8
1	27986065	C	T	0.566666667	1	0.533333333	0.72
1	27986212	C	T	1	0.571428571	0.966666667	1
1	27986224	T	C	0.933333333	0.5	0.966666667	0.88
1	27986272	C	T	0.551724138	0.071428571	0.633333333	0.44
1	27986275	A	G	0.551724138	0	0.433333333	0.28
1	27986278	T	C	0.551724138	0	0.4	0.28
1	27986326	T	C	0.896551724	0.464285714	0.733333333	0.56
1	27986335	C	A	0.517241379	0	0.4	0.24
1	27986591	T	G	0.4	0	0.4	0.16
1	27986597	C	G	1	0.428571429	0.966666667	0.96
1	27986693	A	T	1	0.642857143	0.933333333	0.8
1	27986917	A	G	0.733333333	0.25	0.666666667	0.56
1	27987421	C	T	0.966666667	0.571428571	0.966666667	0.8
1	27987826	T	C	0.333333333	0	0.233333333	0.32
1	27987970	A	G	0.3	0.75	0.1	0.24
1	27988078	T	C	0.533333333	1	0.5	0.72
1	27988362	G	A	1	0.642857143	1	1
3	10627271	G	A	0.384615385	0.821428571	0.4	0.72
4	9371777	G	A	0.230769231	1	0.033333333	0.56
4	9372367	C	G	1	0.16	0.966666667	0.44
4	9372748	G	C	1	0.642857143	1	0.88
4	9372922	C	G	1	0.642857143	0.966666667	0.64
4	9373170	T	C	1	0.392857143	0.966666667	0.8
4	9373604	C	G	1	0.5	0.966666667	0.48
4	9374249	G	A	0.148148148	0.52	0.033333333	0.16
4	9751867	T	A	1	0.178571429	1	0.88
4	9752062	C	T	0.6	0	0.633333333	0.28
4	9752261	C	T	0.586206897	1	0.666666667	0.68
4	9752353	A	T	0.482758621	0	0.466666667	0.2
4	9752357	G	A	0.633333333	1	0.666666667	0.68
4	9752504	C	T	1	0	0.933333333	0.48
4	9752759	A	G	0.6	1	0.7	0.8
4	9752891	C	T	1	0.535714286	0.966666667	0.68
4	9753145	T	C	0.6	0	0.533333333	0.2
7	11995839	C	T	1	0.642857143	1	0.96
7	11995852	T	C	1	0	0.666666667	0.48
7	11995926	G	A	0.740740741	0.321428571	0.633333333	0.56

7	12176292	A	G	0.551724138	0	0.566666667	0.32
7	12240065	C	A	1	0.5	0.866666667	0.64
7	12240195	G	A	0.321428571	1	0.566666667	0.48
7	12240209	A	C	1	0.571428571	0.9	0.92
7	12240236	G	C	1	0.571428571	0.9	0.88
7	12251641	T	C	0.566666667	0	0.666666667	0.36
7	12304264	A	G	0.344827586	0	0.466666667	0.56
7	12316423	A	T	0.9	0.464285714	0.7	0.68
15	4645258	G	C	0.633333333	1	0.7	0.8
15	4645594	C	A	0.962962963	0.333333333	0.933333333	0.76
15	4645607	C	T	0.464285714	0.035714286	0.266666667	0.16

Table S5. Differentiated SNPs in Protein Coding Regions. SNPs within protein coding regions of genes proximal to signals of selection on European (EUR) ancestry in Puerto Rican (PR) honey bees. Down sampled to include only SNPs with substantial variation between the African (AFR) and European panels where the Puerto Rican allele frequency is European-like.

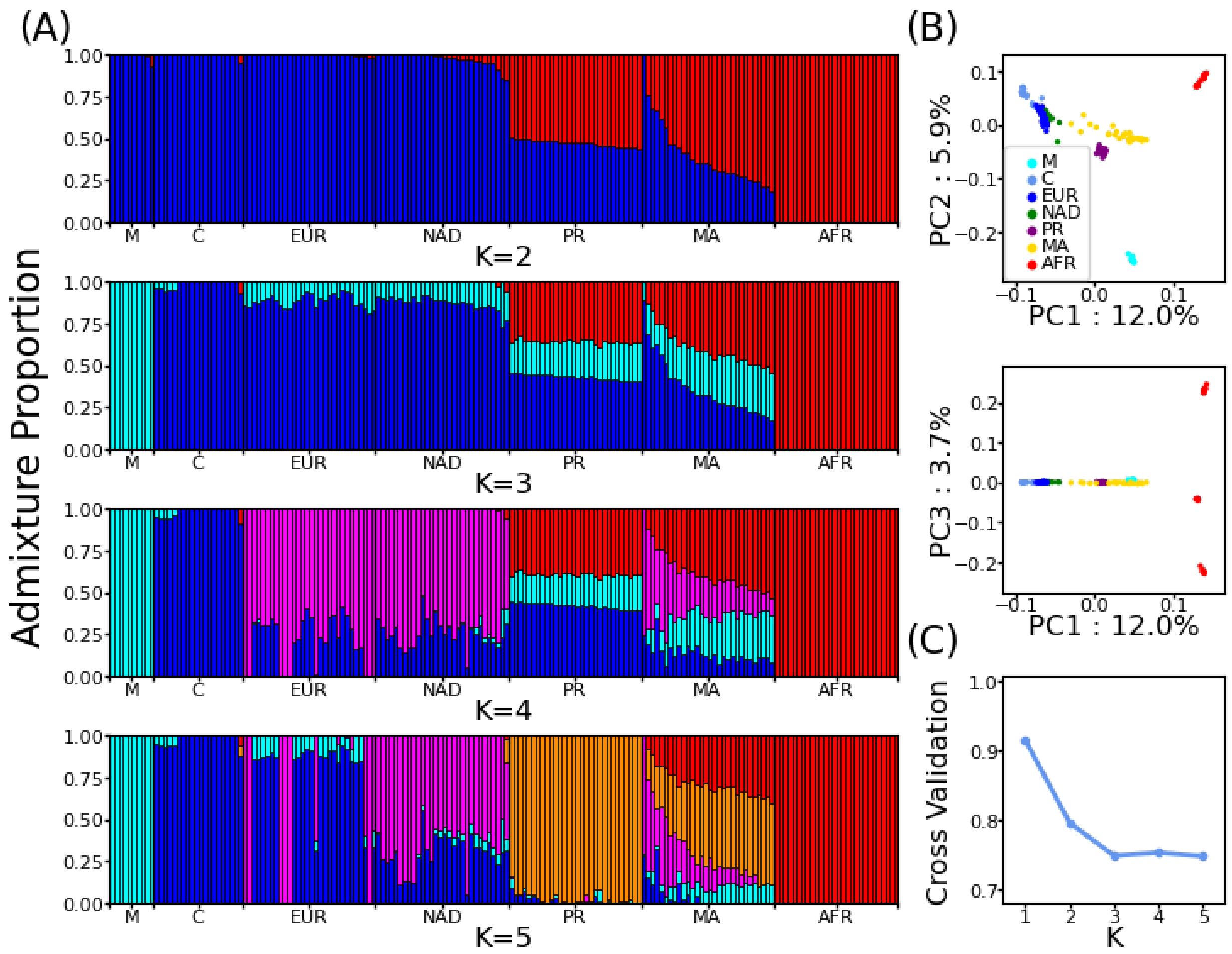

Figure S1. Population structure of M-lineage (M), C-lineage (C), European Domestic (EUR), North American Domestic from Illinois (NAD), Puerto Rican (PR), Mesoamerican (MA) and African (AFR) honey bees. (A) ADMIXTURE (K=2,3,4,5) results of M, C, EUR, NAD, PR, MA and AFR honey bees. For K=2, NAD, PR and MA populations show both African-like ancestry (Red) and European-like ancestry (blue) with an F_{st} of 0.315. For K=3, European-like ancestry subdivides (blue and cyan). At K=3 the F_{st} of blue and cyan (0.429), blue and red (0.542), red and cyan (0.440). At K=4, the results become inconsistent with known population structure, and cross-validation error increases, indicating overfitting. For K=5, AHB ancestry (orange) becomes distinct, consistent with previous structural analyses (Nelson et al. 2017). (B) Principal component analysis (PC1 vs PC2, PC1 vs PC3, and PC1 vs PC4) of genotype data for EUR (blue), NA (green), PR (purple), MA (gold), and AFR (red) honey bees. The first component (PC1), which explains 15.1% of the variance, shows clusters for the EUR and AFR populations with 3 clusters for NAD, PR, MA falling along this axis. The second and third components (PC2 and PC3), explaining 5.8% and 5.0% of the variance respectively, demonstrate the limited structure within the AFR population. The fourth component (PC4), accounting for 2.8% of the variance, shows minimal differentiation of PR and MA populations from EUR,NAD, and AFR populations. (C) Cross-validation using ADMIXTURE results show a shoulder at K=2 with slight improvement for K=3 and K=4, consistent with the subdivision of African-like and AHB ancestries. At K=5, cross-validation error increases, indicating overfitting.

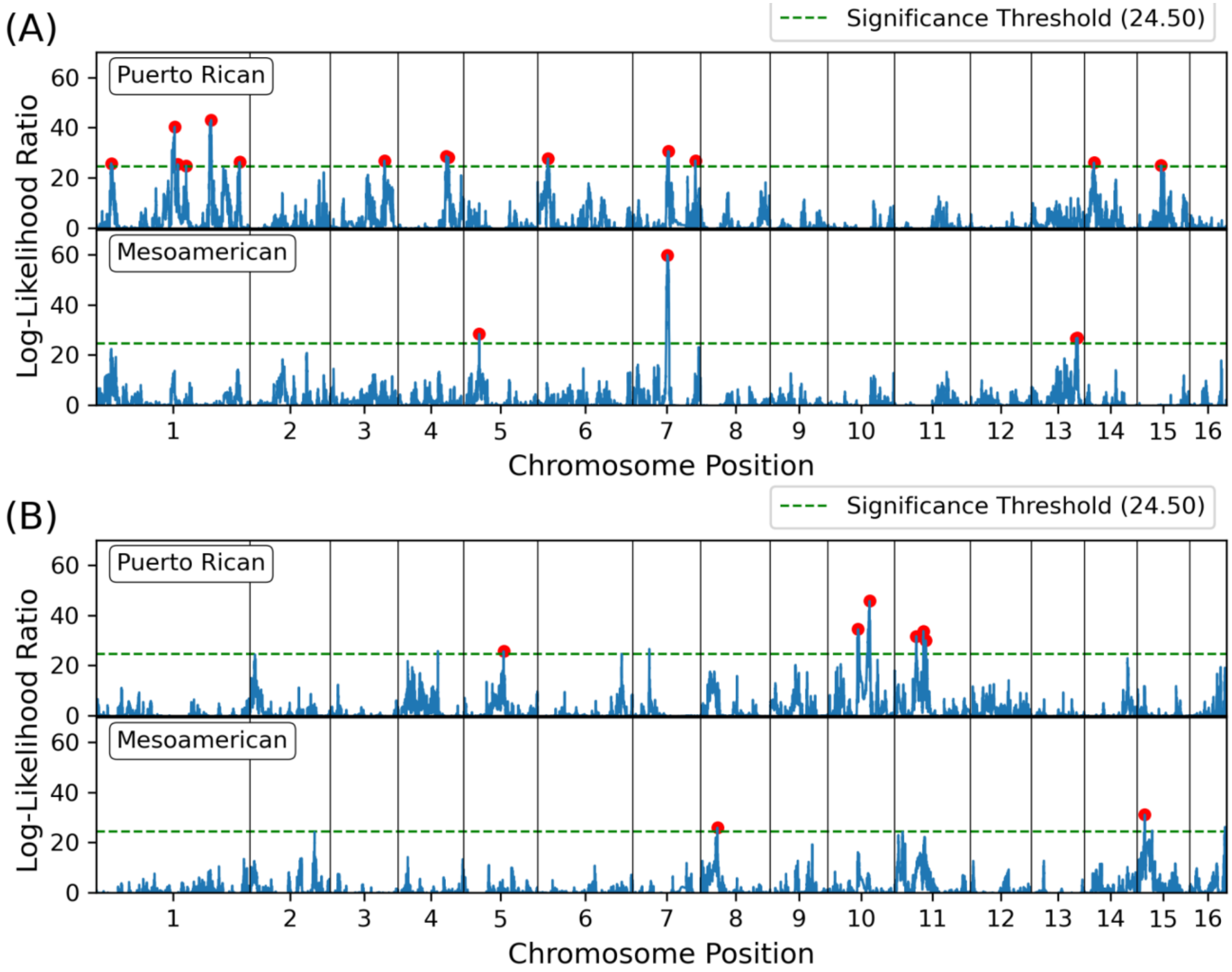

Figure S2. Genome wide signals for ancestry-specific selection on European-like and African-like Ancestry. (A) Log likelihood-ratios for selection on European-like ancestry in Puerto Rican and Mesoamerican honey bees. (B) Log likelihood-ratios for selection on African-like ancestry in Puerto Rican and Mesoamerican honey bees. Vertical black lines indicate chromosome boundaries.

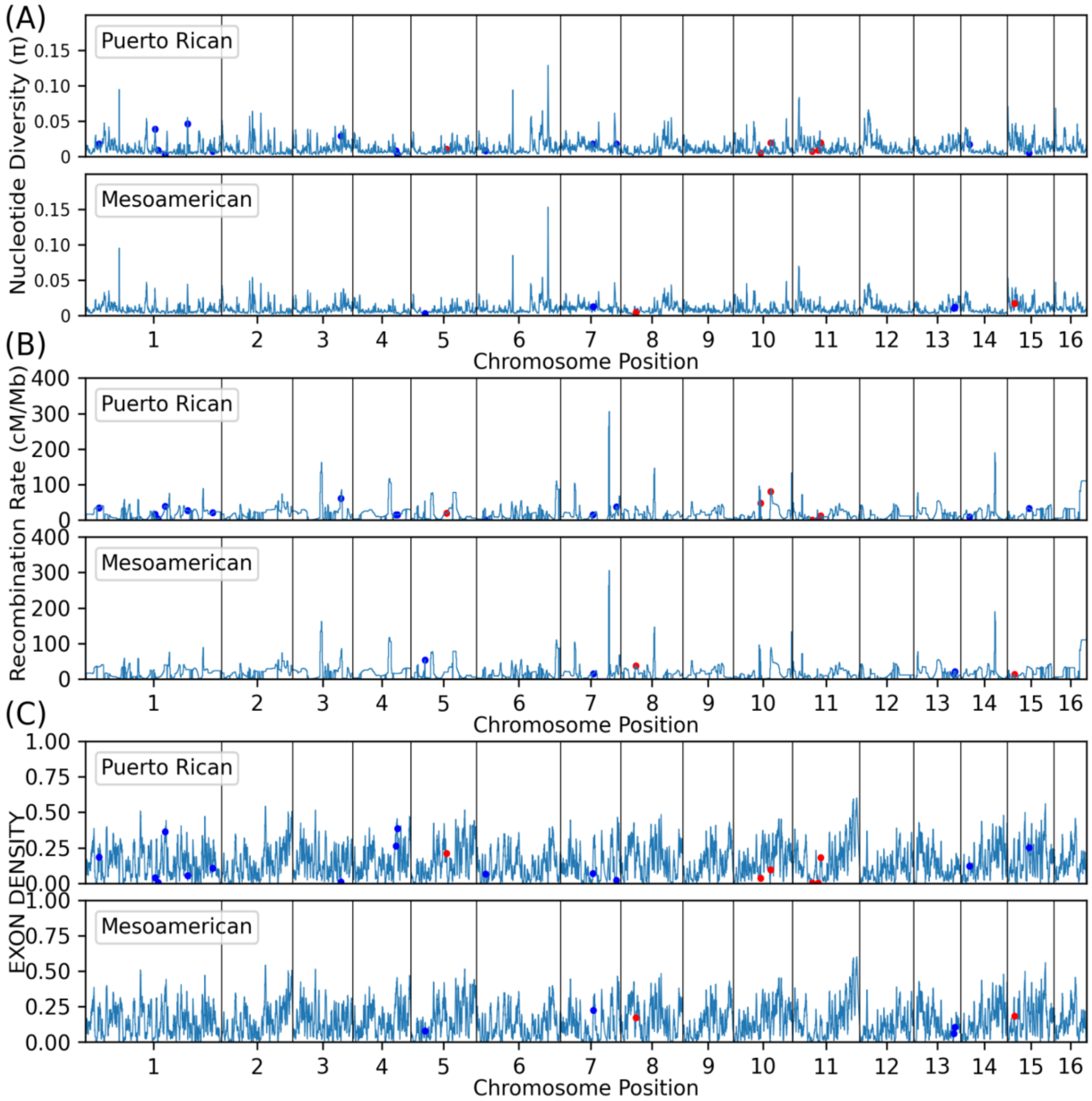

Figure S3. Nucleotide Diversity (π), Recombination Rates and Exon Density. Genome wide nucleotide diversity in Puerto Rican and Mesoamerican honey bees. Calculated from site frequency spectra in 100 KB windows around AIMs. The sites with signals of ancestry selection are shown for African-like ancestry (red) and European-like ancestry (blue). Recombination Rates (cM/Mb) and Exon Density as the proportion of exons in 100 KB windows around AIMs. The sites with signals of ancestry selection in Puerto Rican and Mesoamerican Honeybees are shown for African-like ancestry (red) and European-like ancestry (blue). Vertical black lines indicate chromosome boundaries.

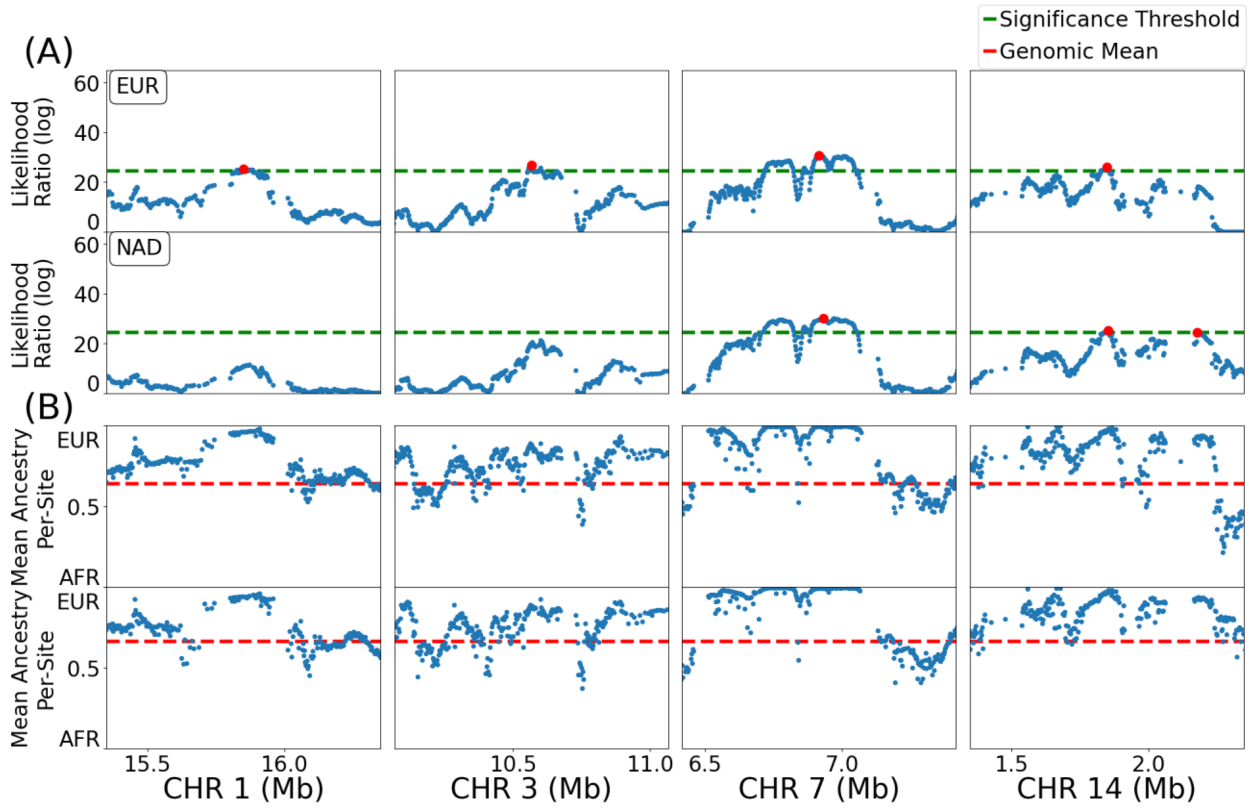

Figure S4. Comparing EHB and NA parental panels on Selection for European-like ancestry. (A) Log likelihood-ratios from AHMM-S and mean ancestry per site from Ancestry_HMM for selection on European ancestry in Puerto Rican honey bees at sites on chromosome 1, chromosome 3, chromosome 7, and chromosome 14. The genome-wide Pearson's correlation coefficient (r) for LAI was 0.8544 and was statistically significant (p-value < 0.0001). (B) Log likelihood-ratios from AHMM-S and mean ancestry per site from Ancestry_HMM for selection on North American ancestry in Puerto Rican honey bees on the same regions of chromosome 1, chromosome 3, chromosome 7, and chromosome 14. The peaks on chromosome 1 and 3 are not over the significance threshold when using North American honey bees as the reference panel, consistent with reduced power as a consequence of introgressed African ancestry.

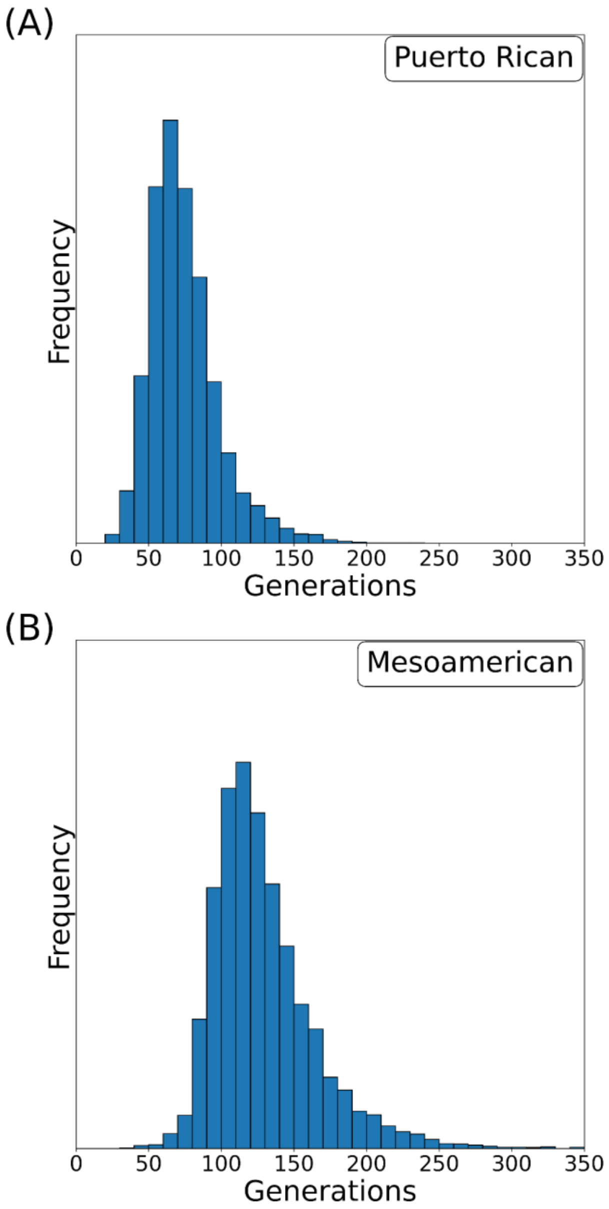

Figure S5. Histogram of Ancestry_HMM Calculated Time Since Admixture: Ancestry_HMM estimate of the number of generations since admixture through bootstrapping 10,000 times. To account for the varying sizes of chromosomes, the number of bootstraps for each chromosome was made proportional to its size in morgans and randomly sampled windows that covered 10% of each chromosome's AIMs. (A) Results of Ancestry_HMM for Puerto Rican honey bees with mean 74.22 generations since admixture. (B) Results of Ancestry_HMM for Mesoamerican honey bees with mean of 129.26 generations since admixture.

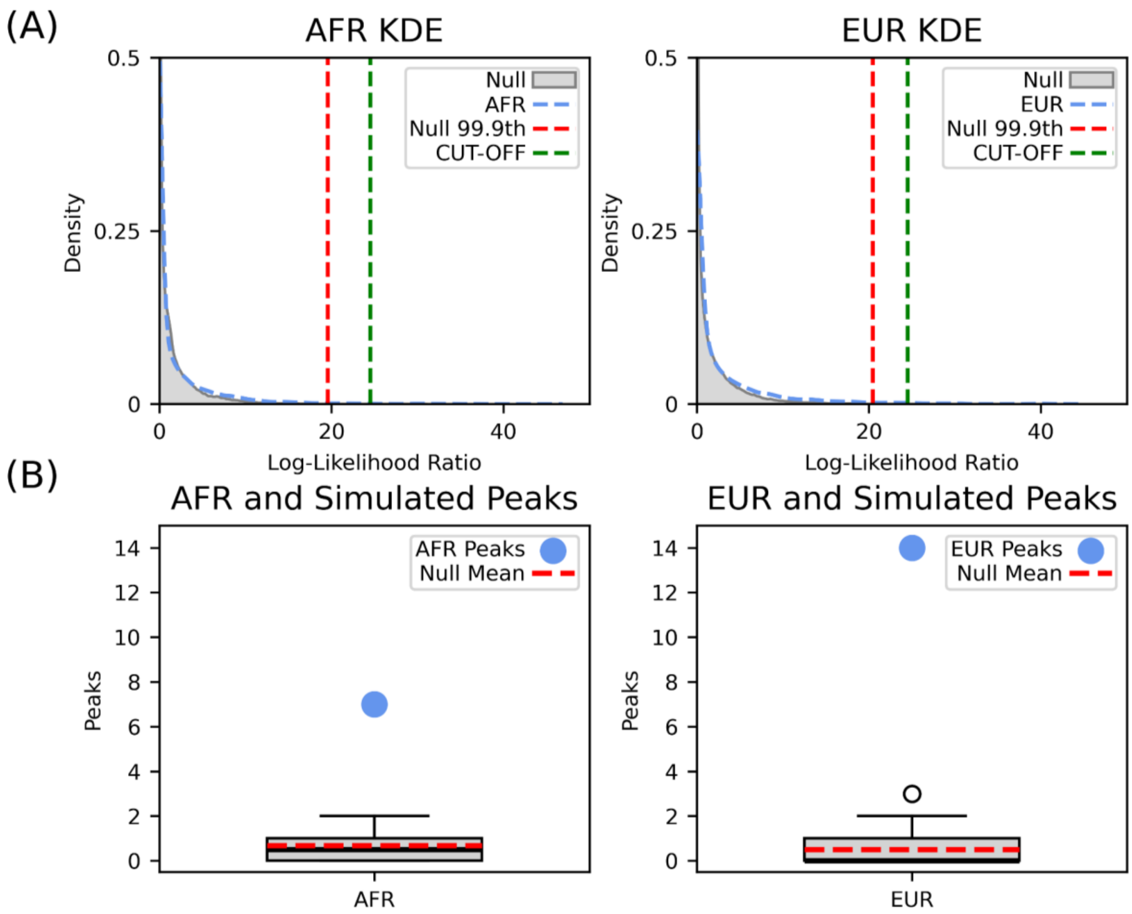

Figure S6. Observed likelihood ratios for selection on EUR and AFR ancestry compared to a null distribution of likelihood ratios under neutrality. (A) Kernel density estimates (KDE) showing the right tail of observed log-likelihood ratios for ancestry-specific selection (blue) compared to the null distribution from 100 neutral simulations (gray) in AFR and EUR ancestry panels. The 99.9th percentile of the null (red; 19.94 for AFR, 20.49 for EUR) is significantly below the genome-wide significance threshold (green; 24.50) used to identify peaks of selection. Both populations show enrichment for high log-likelihood ratios relative to the null. (B) Boxplots summarize the number of peaks identified per simulated genome above the cutoff for each population. The simulated mean is shown as a horizontal line, and the observed peak count is overlaid as a blue dot. The cutoff threshold predicts a mean of 0.67 and 0.51 false discoveries per genome for AFR and EUR, respectively. The simulated demographic model produced an mean estimated EUR ancestry proportion of 0.658 and a time since admixture of 69.4 generations, closely matching empirical estimates for the Puerto Rican population.

References

Avalos A et al. 2017. A soft selective sweep during rapid evolution of gentle behaviour in an Africanized honeybee. Nat. Commun. 8:1550. doi: 10.1038/s41467-017-01800-0.

Fuller ZL et al. 2015. Genome-wide analysis of signatures of selection in populations of African honey bees (Apis mellifera) using new web-based tools. BMC Genomics. 16:518. doi: 10.1186/s12864-015-1712-0.

Galbraith DA, Yang X, Niño EL, Yi S, Grozinger C. 2015. Parallel Epigenomic and Transcriptomic Responses to Viral Infection in Honey Bees (Apis mellifera) Schneider, DS, editor. PLOS Pathog. 11:e1004713. doi: 10.1371/journal.ppat.1004713.

Georgala PA, Carr CB, Price DJ. 2011. The role of Pax6 in forebrain development. Dev. Neurobiol. 71:690–709. doi: 10.1002/dneu.20895.

Ji Y et al. 2020. Gene reuse facilitates rapid radiation and independent adaptation to diverse habitats in the Asian honeybee. Sci. Adv. 6:eabd3590. doi: 10.1126/sciadv.abd3590.

Kim K, Kim JH, Kim YH, Hong S-E, Lee SH. 2018. Pathway profiles based on gene-set enrichment analysis in the honey bee Apis mellifera under brood rearing-suppressed conditions. Genomics. 110:43–49. doi: 10.1016/j.ygeno.2017.08.004.

Kim KC et al. 2014. Pax6-Dependent Cortical Glutamatergic Neuronal Differentiation Regulates Autism-Like Behavior in Prenatally Valproic Acid-Exposed Rat Offspring. Mol. Neurobiol. 49:512–528. doi: 10.1007/s12035-013-8535-2.

Kozmik Z. 2005. Pax genes in eye development and evolution. Curr. Opin. Genet. Dev. 15:430–438. doi: 10.1016/j.gde.2005.05.001.

Macdonald R, Wilson SW. 1997. Distribution of Pax6 protein during eye development suggests discrete roles in proliferative and differentiated visual cells. Dev. Genes Evol. 206:363–369. doi: 10.1007/s004270050065.

Maleszka R, Helliwell P, Kucharski R. 2000. Pharmacological interference with glutamate re-uptake impairs long-term memory in the honeybee, Apis mellifera. Behav. Brain Res. 115:49–53. doi: 10.1016/S0166-4328(00)00235-7.

Nelson RM, Wallberg A, Simões ZLP, Lawson DJ, Webster MT. 2017. Genomewide analysis of admixture and adaptation in the Africanized honeybee. Mol. Ecol. 26:3603–3617. doi: 10.1111/mec.14122.

Parker R et al. 2012. Correlation of proteome-wide changes with social immunity behaviors provides insight into resistance to the parasitic mite, Varroa destructor, in the honey bee (Apis mellifera). Genome Biol. 13:R81. doi: 10.1186/gb-2012-13-9-r81.

Paula GM, Da Silva Menegasso AR, dos-Santos-Pinto JRA, Malaspina O, Palma MS. 2024. Profiling the neuroproteomics of honeybee brain: A clue for understanding the role of neuropeptides in the modulation of aggressivity. J. Proteomics. 295:105089. doi: 10.1016/j.jprot.2024.105089.

Petley-Ragan LM, Ardiel EL, Rankin CH, Auld VJ. 2016. Accumulation of Laminin Monomers in *Drosophila* Glia Leads to Glial Endoplasmic Reticulum Stress and Disrupted Larval Locomotion. J. Neurosci. 36:1151–1164. doi: 10.1523/JNEUROSCI.1797-15.2016.

Pozzi A, Yurchenco PD, Iozzo RV. 2017. The nature and biology of basement membranes. Matrix Biol. 57–58:1–11. doi: 10.1016/j.matbio.2016.12.009.

Ross EM, Wilkie TM. 2000. GTPase-Activating Proteins for Heterotrimeric G Proteins: Regulators of G Protein Signaling (RGS) and RGS-Like Proteins. Annu. Rev. Biochem. 69:795–827. doi: 10.1146/annurev.biochem.69.1.795.

Si A, Helliwell P, Maleszka R. 2004. Effects of NMDA receptor antagonists on olfactory learning and memory in the honeybee (Apis mellifera). Pharmacol. Biochem. Behav. 77:191–197. doi: 10.1016/j.pbb.2003.09.023.

Van Heyningen V. 2002. PAX6 in sensory development. Hum. Mol. Genet. 11:1161–1167. doi: 10.1093/hmg/11.10.1161.

Vieira J et al. 2021. Molecular underpinnings of the early brain developmental response to differential feeding in the honey bee Apis mellifera. Biochim. Biophys. Acta BBA - Gene Regul. Mech. 1864:194732. doi: 10.1016/j.bbagrm.2021.194732.

Wang K et al. 2020. Epigenetic Modifications May Regulate the Activation of the Hypopharyngeal Gland of Honeybees (Apis Mellifera) During Winter. Front. Genet. 11:46. doi: 10.3389/fgene.2020.00046.

Wu T et al. 2023. Interaction of chlorothalonil and Varroa destructor on immature honey bees rearing in vitro. Sci. Total Environ. 904:166302. doi: 10.1016/j.scitotenv.2023.166302.

Zhao Y et al. 2019. The Dynamics of Deformed Wing Virus Concentration and Host Defensive Gene Expression after Varroa Mite Parasitism in Honey Bees, Apis mellifera. Insects. 10:16. doi: 10.3390/insects10010016.
